# It’s a match: use of the radionuclide theranostic pair ^133^La/^225^Ac for the radiopharmacological characterization of EGFR-targeted single-domain antibodies

**DOI:** 10.1186/s41181-025-00354-7

**Published:** 2025-06-19

**Authors:** Johanna Trommer, Martin Ullrich, Falco Reissig, Santiago Andres Brühlmann, Anne-Kathrin Nitt-Weber, Zbynek Novy, Katarina Hajduova, Daniela Kurfurstova, Romana Hendrychova, Jan Bouchal, Milos Petrik, Christin Neuber, Wiebke Sihver, Sven Stadlbauer, Jens Pietzsch, Martin Kreller, Klaus Kopka, Constantin Mamat, Kristof Zarschler

**Affiliations:** 1 Helmholtz-Zentrum Dresden-Rossendorf, Institute of Radiopharmaceutical Cancer Research, Dresden, Germany; 2 Institute of Molecular and Translational Medicine, Faculty of Medicine and Dentistry, Palacký University, Olomouc, Czech Republic; 3 Czech Advanced Technology and Research Institute, Palacký University, Olomouc, Czech Republic; 4 Institute of Clinical and Molecular Pathology, Faculty of Medicine and Dentistry, Palacký University Olomouc, Olomouc, Czech Republic; 5 Laboratory of Experimental Medicine, University Hospital, Olomouc, Czech Republic; 6 School of Science, Faculty of Chemistry and Food Chemistry, Technische Universität Dresden, Dresden, Germany; 7 National Center for Tumor Diseases (NCT) Dresden, University Hospital Carl Gustav Carus, Fetscherstraße 74, 01307 Dresden, Germany; 8 German Cancer Consortium (DKTK), Partner Site Dresden, Fetscherstraße 74, 01307 Dresden, Germany

**Keywords:** ^133^La, ^225^Ac, Macropa, Targeted alpha therapy TAT, Single-domain antibody, Theranostics, Biodistribution, Pharmacokinetics, Positron emission tomography

## Abstract

**Background:**

Targeted alpha therapy represents an advanced and rapidly evolving form of precision cancer treatment with increasing importance in recent years. The alpha-emitter ^225^Ac plays a key role in this clinical development due to its attractive physical and chemical properties. In this context, the macropa chelator has favorable characteristics in terms of labeling conditions and complex stability, making its derivatives exceptionally appealing for ^225^Ac-labeling of heat-sensitive biomolecules. However, preclinical evaluation of such ^225^Ac-containing molecules and comprehensive assessment of their pharmacokinetics, dosimetry and radiobiology necessitate a suitable diagnostic counterpart. Due to its attractive radiation properties, ^133^La represents an adequate positron-emitting radionuclide to form a matched pair with ^225^Ac for macropa-based radiopharmaceuticals. Herein, we describe the preparation and radiopharmacological characterization of macropa-functionalized, ^133^La/^225^Ac-labeled single-domain antibodies (sdAbs) targeting the epidermal growth factor receptor (EGFR) to demonstrate the general suitability of this theranostic pair of radionuclides.

**Results:**

The synthesis of a clickable, bicyclononyne-modified macropa chelator and its site-specific conjugation to azide-modified, monovalent and biparatopic sdAbs is presented. Subsequent labeling at room temperature (rt) for 15 min resulted in molar activities of 30 MBq/nmol for ^133^La and 0.5 MBq/nmol for ^225^Ac, respectively. In vitro studies using the ^133^La-labeled sdAbs revealed comparable binding characteristics, but an enhanced cellular internalization of the biparatopic variant compared to its monovalent counterparts. This increased uptake consequently resulted in higher cytotoxicity of the ^225^Ac-labeled biparatopic conjugate. In vivo PET imaging of the ^133^La-labeled conjugates indicated comparable uptake and retention of the mono- and biparatopic variants in liver and kidneys, with the former showing slightly higher tumor accumulation. Ex vivo biodistribution studies conducted with ^225^Ac-labeled conjugates largely confirmed the findings obtained by PET imaging, albeit with a marginally higher tumor accumulation of the biparatopic ^225^Ac-radioimmunoconjugate. Final histological examinations of tumor and kidney tissues showed DNA damage in the renal cortex of the ^225^Ac-radioimmunoconjugate-treated mice, but no differences in the number of γ-H2AX-positive cells in the corresponding tumor tissues could be detected.

**Conclusions:**

We present a comprehensive study on the theranostic application of ^133^La and ^225^Ac for antibody-based biomolecules and lay the foundation for the future application of this matched pair of radionuclides towards labeling of heat-sensitive, macropa-functionalized radiopharmaceuticals in general.

**Supplementary Information:**

The online version contains supplementary material available at 10.1186/s41181-025-00354-7.

## Background

Matched theranostic radionuclide pairs are used in nuclear medicine for the labeling of molecular target vectors, whereby both the diagnostic and the therapeutic radionuclides should have similar chemical properties ideally originating from the same element (Kelkar and Reineke 2011; Vahidfar et al. 2021; Currie and Rohren 2025). Labeling of the vector molecule with positron- or photon-emitting radionuclides enables a diagnostic application using positron emission tomography (PET) or single-photon emission computed tomography (SPECT) imaging, while the combination with particle-emitting radionuclides provides the basis for targeted radionuclide therapy (TRNT) (Bodei et al. 2022; Burkett et al. 2023). This emerging strategy involves precise elimination of malignancies with α, β^−^, or Auger electron (AE) emitters, which is achieved by the internal irradiation of the tumor cells with their particle emissions and the deposition of highly ionizing radiation in the tumor, whereby the surrounding healthy tissue is spared (White et al. 2021; Lepareur et al. 2023). Targeted alpha therapy (TAT) plays a key role in this context, as α-emitters are characterized by a high linear energy transfer (LET) emission path of 50–100 μm, thus giving them an effective range of a few cell diameters and, therefore, minimizes irradiation of the neighboring healthy tissue (Kozempel et al. 2018; Makvandi et al. 2018; King et al. 2021; Coll et al. 2023). Due to its almost ideal physical properties, which are particularly evident in its half-life of 9.9 days and a cascade decay with four α-particle emissions, ^225^Ac has demonstrated exceptional efficacy and enormous potential in TAT (Morgenstern et al. 2018; Robertson et al. 2018; Roscher et al. 2020; Mourtada et al. 2023; Hooijman et al. 2024). Consequently, it is highly attractive for the treatment of micrometastatic cancers and small tumors as well as eradication of hematologic cancers and residual tumor cells (Jurcic 2019; Jang et al. 2023; Bidkar et al. 2024; Miederer et al. 2024). The risk of severe off-target toxicity to nontarget tissues represents the downside of the high potency of ^225^Ac in TAT and requires an in-depth preclinical characterization as well as a comprehensive understanding of the pharmacokinetics, dosimetry and radiobiology of ^225^Ac-based radiopharmaceuticals (Sgouros et al. 2018; Castillo Seoane et al. 2020; Tronchin et al. 2022). Therefore, a corresponding paired imaging surrogate is required to address these crucial issues in more detail, with positron emitters being particularly suited to perform dosimetry calculations (Nelson et al. 2023). In this respect, various lanthanum radionuclides have been investigated recently, as this metal represents an attractive surrogate for actinium with comparable ionic radii and similar coordination chemistry (Nelson et al. 2022a, b). Among the different positron-emitting radiolanthanum isotopes, in particular ^133^La shows high potential for precision PET imaging applications due to its reliable, high-yield, cyclotron-based production, efficient separation and attractive decay properties (Nelson et al. 2020, 2022a, b; Brühlmann et al. 2024). Since the efficient complexation of ^133^La (and ^225^Ac) with the popular standard macrocyclic complexing agent DOTA (1,4,7,10-tetraazacyclododecane-1,4,7,10-tetraacetic acid) requires elevated reaction temperatures, its use is limited to thermostable vector molecules (Price and Orvig 2014; Morgenstern et al. 2021). The 18-membered macrocyclic chelator macropa (*N*,*N*’-bis[(6-carboxy-2-pyridyl)methyl]-4,13-diaza-18-crown-6), in contrast, enables highly stable and rapid complexation with trivalent ^13x^La and ^225^Ac cations under mild conditions at ambient temperature, allowing direct radiolabeling of heat-sensitive molecular targeting vectors including antibodies and antibody fragments (Thiele et al. 2017; Yang et al. 2020; Kadassery et al. 2022; Blei et al. 2023).

Herein, we present a comprehensive radiopharmacological characterization of site-specifically modified and ^133^La/^225^Ac-labeled sdAbs targeting the human EGFR. This investigation aims to assess the therapeutic potential of this specific theranostic matched pair of radionuclides in combination with a clickable macropa derivative and heat-sensitive biomolecules for addressing tumor-specific membrane proteins in both in vitro and in vivo models. With their high binding specificity, nanomolar binding affinity and low immunogenic profile, sdAbs generated from camelid heavy-chain-only antibodies possess invaluable attributes as molecular probes for imaging and targeted radionuclide therapy (Jovcevska and Muyldermans 2020; Hurley et al. 2023). Compared to full-length antibodies, their considerably smaller size promotes better tumor penetration, shorter blood circulation time and rapid renal clearance. Additional crucial advantages of sdAbs include their efficient production in prokaryotic or eukaryotic expression systems as well as their straightforward engineering for the generation of bivalent, biparatopic and bispecific formats (Muyldermans 2013; Akiba and Tsumoto 2020). Bivalent monospecific constructs consist of two identical antigen-binding domains with increased functional binding affinity (avidity), while biparatopic monospecific constructs result from the tandem assembly of two different sdAbs recognizing different epitopes of the same antigen (Niquille et al. 2024). Due to its crucial involvement in several human epithelial malignancies and its enormous importance as an anticancer drug target (Wang 2017; Levantini et al. 2022), the antibody-based targeting of EGFR has been the focus of multiple scientific studies leading to the identification and characterization of numerous EGFR-specific sdAbs, including several multivalent and multispecific constructs (Gainkam et al. 2008, 2011a, b; Huang et al. 2008; Roovers et al. 2011; Heukers et al. 2013; Zarschler et al. 2013; Li et al. 2015; Tripathy and Pande 2024). From this existing portfolio, we select two monovalent anti-EGFR sdAbs to engineer a biparatopic variant and, subsequently, introduce a macropa chelator regioselectively. This procedure provides the basis for subsequent radiolabeling with the theranostic pair ^133^La/^225^Ac and the detailed preclinical evaluation of the radioimmunoconjugates with regard to stability, cell binding and in vitro toxicity as well as pharmacokinetics, biodistribution and tumor targeting.

## Results

**Synthesis of BCN-PEG**_**5**_**-mcp.** Previous studies already demonstrated that macropa and its derivatives are ideally suited for the stable complexation of ^225^Ac (Thiele et al. 2017; Reissig et al. 2021, 2022) and its diagnostic counterpart ^133^La (Nelson et al. 2020, 2022a, b; Brühlmann et al. 2022; Blei et al. 2023). Strain-promoted azide-alkyne cycloaddition (SPAAC) was chosen for the covalent attachment of the chelator to the sdAbs to avoid the unfavorable removal of copper when using the Cu-catalyzed version. Bicyclononyne (BCN) is readily available and allows a fast click reaction under mild conditions at ambient temperatures (Dommerholt et al. 2010).

**Fig. 1:**
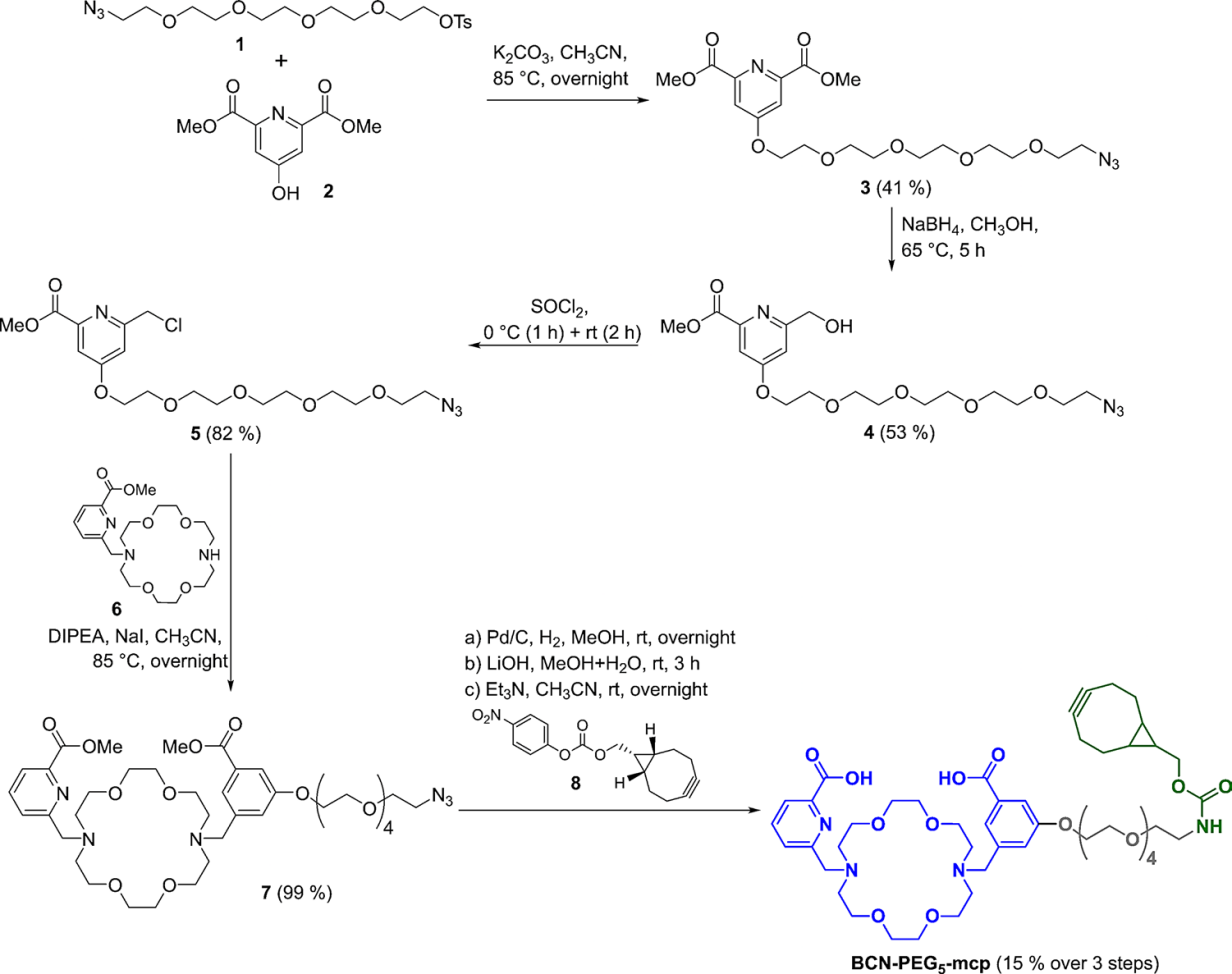
Synthesis pathway for the synthesis of the clickable macropa chelator. The final **compound BCN-PEG**_**5**_**-mcp** consists of the clickable BCN moiety (green) for connection to the azide-functionalized single-domain antibody, the macropa chelator (blue) for radiolabeling with ^133^La and ^225^Ac, and the PEG-linker (grey)

Starting from dimethyl 4-hydroxypyridine-2,6-dicarboxylate (**2**) which was reacted with the short azide-functionalized PEG_5_ linker **1**, a straightforward synthesis was developed followed by the reduction of one of the carboxylates and chlorination of the hydroxy group to yield the pyridine side arm **5** (Fig. 1). Compound **5** was subsequently reacted with aza-crown ether **6** to form macropa compound **7**. This was followed by incorporation of the BCN moiety **8**, achieved through azide reduction to an amine and ester saponification under basic conditions, ultimately yielding the bicyclononyne-modified macropa chelator **BCN-PEG**_**5**_**-mcp**. Detailed information on the synthesis as well as the ^1^H and ^13^C NMR spectra of the respective compounds are provided in the Supporting Information (Supplemental Figs. 1–15).

**Fig. 2:**
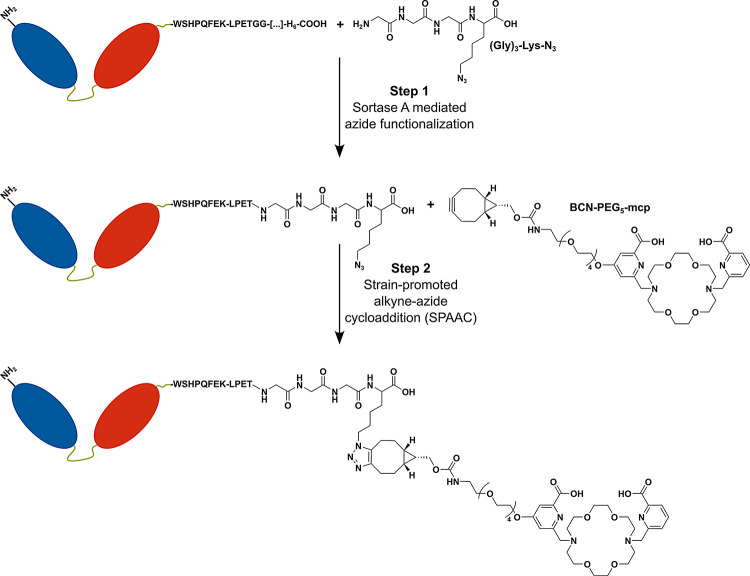
Site-specific modification of single-domain antibodies using a combination of enzyme-mediated bioconjugation and click chemistry. The biparatopic construct depicted here as an example consists of two anti-EGFR single-domain antibody fragments (blue and red) connected via a flexible linker (green). The constructs possess a C-terminal Strep-tag (WSHPQFEK), a Sortase A recognition motif (LPETG) as well as a hexahistidine tag (H_6_). In the first step, an azide functionality is site-specifically introduced using the Sortase substrate (Gly)_3_-Lys-N_3_. The azide-functionalized single-domain antibody reacts in the second step with the bicyclononyne-modified macropa chelator (**BCN-PEG**_**5**_**-mcp**) in a cycloaddition reaction

**Synthesis**,** radiolabeling and stability of the macropa-sdAb conjugates.** The two-step, site-specific attachment of the bicyclononyne-modified macropa chelator **BCN-PEG**_**5**_**-mcp** to the EGFR-specific, monovalent and biparatopic sdAbs is schematically shown in Fig. 2. This procedure requires protein engineering to equip the desired conjugation site at the C-terminal end of the sdAbs with the unique sortase recognition motif. Therefore, the sdAbs were engineered with C-terminal tags consisting of a Strep-tag (WSHPQFEK), the LPETG sortase motif, and a hexahistidine purification tag (H_6_) resulting in the monovalent sdAbs A-Strep-sortag-H_6_ and B-Strep-sortag-H_6_, as well as the biparatopic AB-Strep-sortag-H_6_. The bifunctional linker (Gly)_3_-Lys-N_3_ was then allowed to react with the sdAbs in the presence of sortase A, and the resulting products were purified by affinity chromatography. Finally, the bicyclononyne-modified macropa chelator **BCN-PEG**_**5**_**-mcp** was conjugated with the azide-functionalized sdAbs catalyzed by SPAAC, yielding the single-conjugated products mcp-A, mcp-B and mcp-AB.

**Fig. 3:**
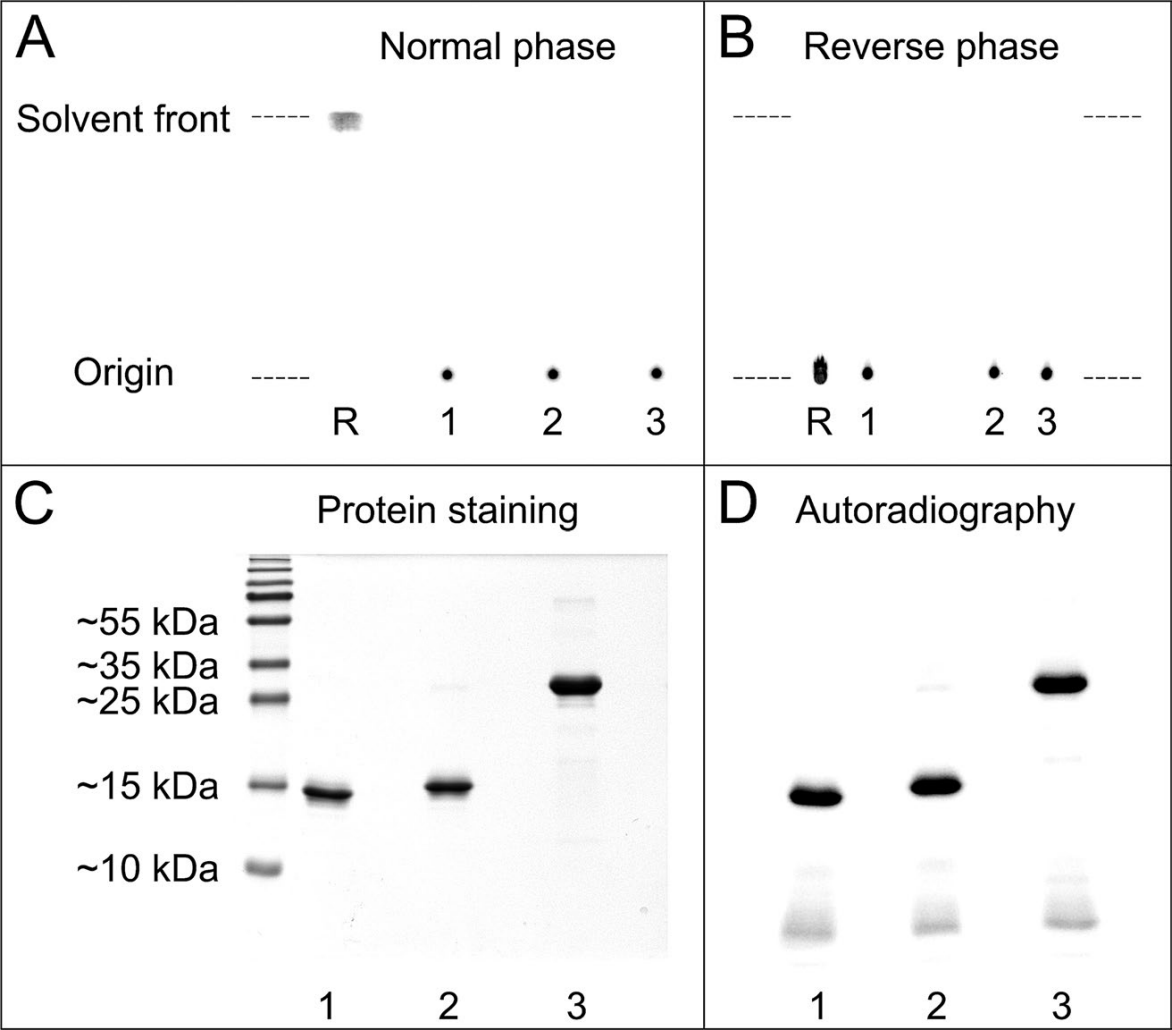
Radio-TLC (**A**, **B**) and radio-SDS-PAGE (**C**, **D**) analysis of ^133^La-labeled, macropa-conjugated sdAbs. In the normal phase TLC system (A), [^133^La]La-mcp-A (1), [^133^La]La-mcp-B (2) and [^133^La]La-mcp-AB (3) remain at the origin (*R*_f_ = 0), whereas free [^133^La]La (R) is complexed by EDTA of the mobile phase and migrates as [^133^La]La-EDTA with the solvent front (*R*_f_ = 1). The reverse phase TLC system (B) was designed to detect traces of radiolabeled **BCN-PEG**_**5**_**-mcp** with an *R*_f_ ~0.7, whereas free [^133^La]La^3+^ (R), [^133^La]La-mcp-A (1), [^133^La]La-mcp-B (2), and [^133^La]La-mcp-AB (3) remain at the origin. Coomassie stained 15% SDS-polyacrylamide gel (C) and autoradiography (D) show bands of [^133^La]La-mcp-A (1, ~ 17.4 kDa), [^133^La]La-mcp-B (2, ~ 18.3 kDa) and [^133^La]La-mcp-AB (3, ~ 32.6 kDa). The presence of minimal traces of free [^133^La]La^3+^ (< 0.5%) is indicated by the observation of faint bands at the bottom of the autoradiographic gel image (D)

The macropa-sdAb conjugates were successfully radiolabeled with ^133^La and ^225^Ac, achieving radiochemical conversions exceeding 99% within 15 min at rt. The resulting molar activities were 30 MBq/nmol for ^133^La and 0.5 MBq/nmol for ^225^Ac, respectively. The ^133^La-labeled immunoconjugates were analyzed by thin-layer chromatography (TLC) and non-reducing sodium dodecyl sulfate-polyacrylamide gel electrophoresis (SDS-PAGE) to verify their purity, integrity and identity (Fig. 3). Corresponding radio-TLC analysis of ^225^Ac-labeled macropa-sdAbs is shown in Supplemental Fig. 16. These radioimmunoconjugates were stable for up to 10 d in both PBS and human serum, retaining > 90% integrity at each time point assayed (Supplemental Fig. 17).

**Fig. 4:**
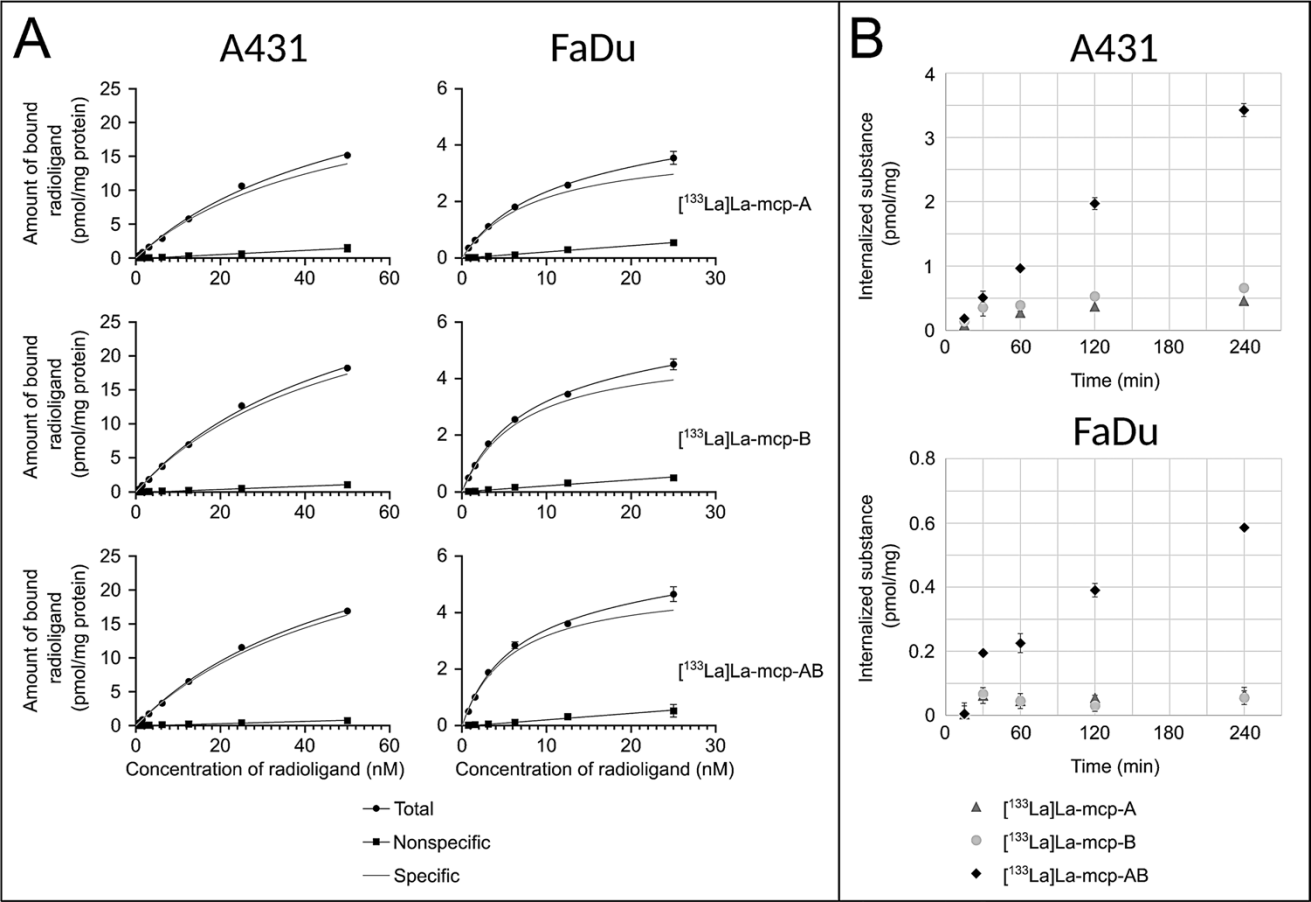
Evaluation of cell binding and internalization of ^133^La-labeled single-domain antibodies. (**A**) Saturation binding curves for the monovalent [^133^La]La-mcp-A and [^133^La]La-mcp-B, respectively, as well as the biparatopic [^133^La]La-mcp-AB single-domain antibodies. Nonspecific binding was determined in the presence of 10 µM unlabeled ligand. Specific binding was calculated as the difference between total and nonspecific binding. (**B**) Time dependent, EGFR-mediated cell uptake of ^133^La-labeled single-domain antibodies after incubation with 10 nM of [^133^La]La-mcp-A, [^133^La]La-mcp-B or [^133^La]La-mcp-AB, respectively, for up to 4 h at 37 °C. Nonspecific binding was determined in the presence of 1 µM unlabeled ligand. Specific internalization was calculated as the difference between total and nonspecific internalization

**Analysis of the EGFR-binding properties.** Cell-binding and internalization studies of ^133^La-labeled macropa-sdAb conjugates were performed using the human epidermoid carcinoma cell line A431 and the squamous carcinoma cell line FaDu characterized by high and moderate EGFR expression, respectively (see supplemental Fig. 18 for comparative, ELISA-based quantification of full-length EGFR in respective cell lysates). Binding of the radioimmunoconjugates to the cells was blocked in the presence of an excess of unlabeled sdAbs confirming their target specificity. The equilibrium dissociation constants (*K*_d_) and maximum binding capacities (B_max_) were determined by saturation binding assays (Table 1; Fig. 4A).

**Table 1:** Summary of in vitro binding characteristics for the different ^133^La-labeled immunoconjugates obtained with EGFR + A431 and FaDu cells.^a^

Ligand	Cell line	K_d_ (nM)^c^	B_max_ (pmol/mg protein)^c^	Specific internalization (%)^b^
[^133^La]La-mcp-A	A431	46.37 (38.98–55.78)	26.83 (24.20–30.07)	22.0
[^133^La]La-mcp-B	A431	48.71 (43.62–54.64)	34.20 (31.99–36.73)	34.5
[^133^La]La-mcp-AB	A431	42.70 (34.50–53.68)	33.22 (29.37–38.16)	59.1
[^133^La]La-mcp-A	FaDu	8.910 (7.669–10.37)	4.032 (3.779–4.316)	15.7
[^133^La]La-mcp-B	FaDu	6.974 (6.215–7.831)	5.051 (4.823–5.298)	14.0
[^133^La]La-mcp-AB	FaDu	5.539 (4.987–6.156)	5.519 (5.307–5.746)	30.3

^a^ One experiment which was performed in triplicate

^b^ After 4 h of incubation at 37 °C with 10 nM of the corresponding radioimmunoconjugate

^c^ 95% confidence interval

The three analyzed, ^133^La-labeled immunoconjugates possess comparable binding characteristics with nanomolar binding affinities for both EGFR-positive cell lines, and no substantial differences in the in vitro receptor binding affinity between [^133^La]La-mcp-A, [^133^La]La-mcp-B and [^133^La]La-mcp-AB were observed.

In addition, the specific time-dependent internalization via receptor-mediated endocytosis into the EGFR-expressing cells was determined (Table 1; Fig. 4B). The two radiolabeled monovalent macropa-sdAb constructs exhibit significantly lower cellular internalization compared to the biparatopic conjugate. After 4 h of incubation with 10 nM of the respective radioimmunoconjugate, A431 cells take up approximately seven times less [^133^La]La-mcp-A (0.46 pmol/mg) and five times less [^133^La]La-mcp-B (0.66 pmol/mg) compared to [^133^La]La-mcp-AB (3.42 pmol/mg). This means that almost 60% of the bound biparatopic construct is taken up by receptor-mediated endocytosis, whereas the internalized fractions of [^133^La]La-mcp-A and of [^133^La]La-mcp-B are 22.0% and 34.5%, respectively. A similar trend is apparent for FaDu cells, although the absolute amounts of internalized substance are lower ranging from 0.07 pmol/mg and 0.05 pmol/mg for [^133^La]La-mcp-A and [^133^La]La-mcp-B, respectively, to 0.59 pmol/mg for [^133^La]La-mcp-AB. The presence of two separate EGFR binding sites in the biparatopic construct obviously leads to an increased receptor-mediated uptake by simultaneous interaction with the different antigen epitopes.

**Fig. 5:**
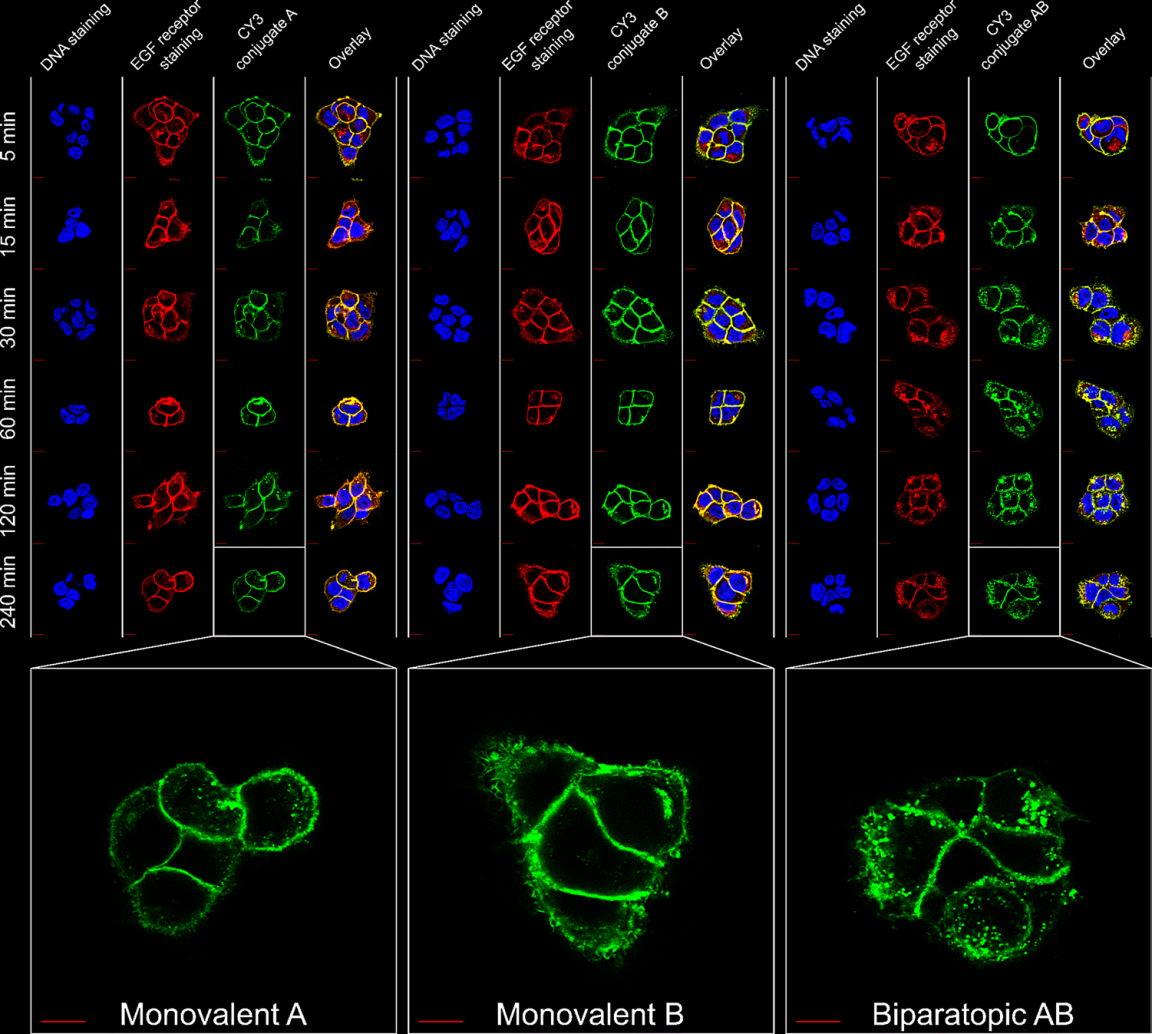
Confocal immunofluorescence microscopy images of A431 cells exposed to the fluorescent-labeled monovalent single-domain antibodies A and B or to the biparatopic variant AB for up to 4 h at 37 °C showing time-dependent, specific binding and colocalization of the conjugates with EGFR. An anti-EGFR Alexa Fluor 647 antibody conjugate was used to confirm EGFR expression. The nuclei were visualized by the DNA binding stain DAPI. Scale bars: 20 μm

The cellular uptake of the sdAbs was further examined by fluorescence microscopy to visualize the differences in their subcellular localization over time (Fig. 5). Confocal microscopy of A431 cells showed uniform association with the cell membrane and co-localization with EGFR already after 5 min of incubation. This membrane localization is largely retained over time for the monovalent constructs and only very little intracellular fluorescence was observed. The biparatopic variant, by contrast, exhibits time-dependent internalization, as indicated by a punctiform, intracellular fluorescence pattern alongside membrane staining.

**Fig. 6:**
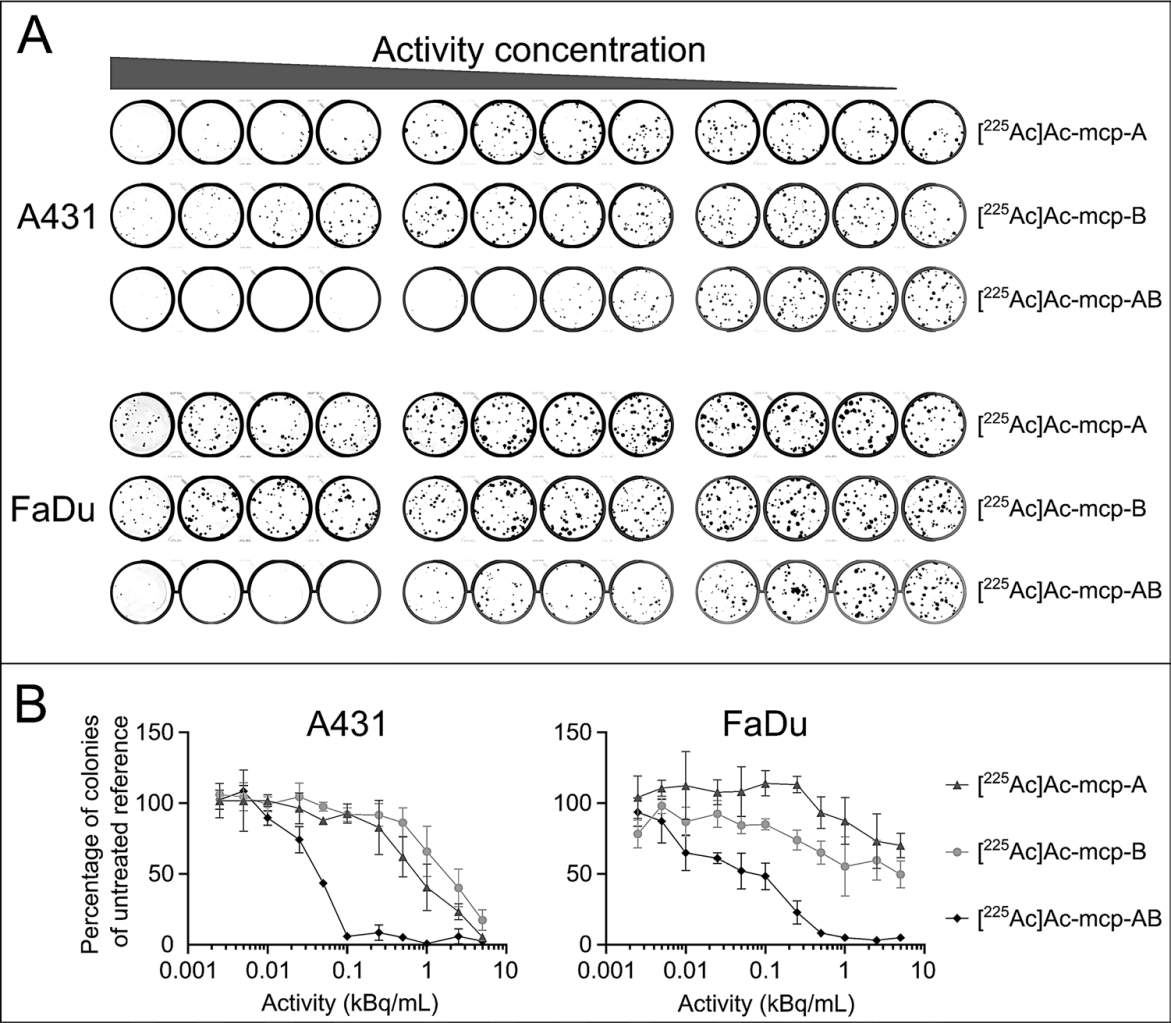
Clonogenic survival of A431 and FaDu cells after treatment with ^225^Ac-labeled single-domain antibodies. Cells were exposed to different activity concentrations of ^225^Ac-labeled single-domain antibody conjugates for 4 h and thereafter supplemented with fresh medium. Seven (A431) or eleven (FaDu) days later, formed colonies were stained, imaged and counted by digital analysis. (**A**) Representative images of crystal violet stained wells after colony-forming assay using A431 and FaDu cells upon exposure to decreasing activity concentrations (5 kBq/mL – 0 kBq/well) of monovalent [^225^Ac]Ac-mcp-A and [^225^Ac]Ac-mcp-B, respectively, or the biparatopic [^225^Ac]Ac-mcp-AB single-domain antibodies. (**B**) Corresponding dose-response curves for clonogenic assay. Data points represent the mean colony number of three samples, normalized to values obtained for the untreated reference samples

**Cytotoxicity of** ^**225**^**Ac-labeled macropa-sdAb conjugates.** Assessment of the clonogenic activity of EGFR + A431 and FaDu cells after exposure to increasing activity concentrations (0–5 kBq/mL) of the monovalent [^225^Ac]Ac-mcp-A and [^225^Ac]Ac-mcp-B or the biparatopic [^225^Ac]Ac-mcp-AB revealed a dose-dependent decrease in colony-forming cells (Fig. 6).

The EGFR-targeted alpha therapy with ^225^Ac-labeled immunoconjugates caused considerable cytotoxicity for both cell lines as determined by the number of evolving colonies. The biparatopic sdAb blocks colony outgrowth of A431 cells by about 50% at an activity concentration of ~ 0.05 kBq/mL with an almost complete inhibition of colony formation observed at a concentration of 0.1 kBq/mL. Its monovalent counterparts [^225^Ac]Ac-mcp-A and [^225^Ac]Ac-mcp-B show an approximately 50% inhibition on colony formation at ~ 0.7 and 2.5 kBq/mL, respectively. Comparable differences in the therapeutic potency of the ^225^Ac-labeled immunoconjugates are observed in FaDu cells, where higher activity concentrations are required to achieve substantial suppression of clonogenic survival due to the lower antigen density on the target cells.

**Fig. 7:**
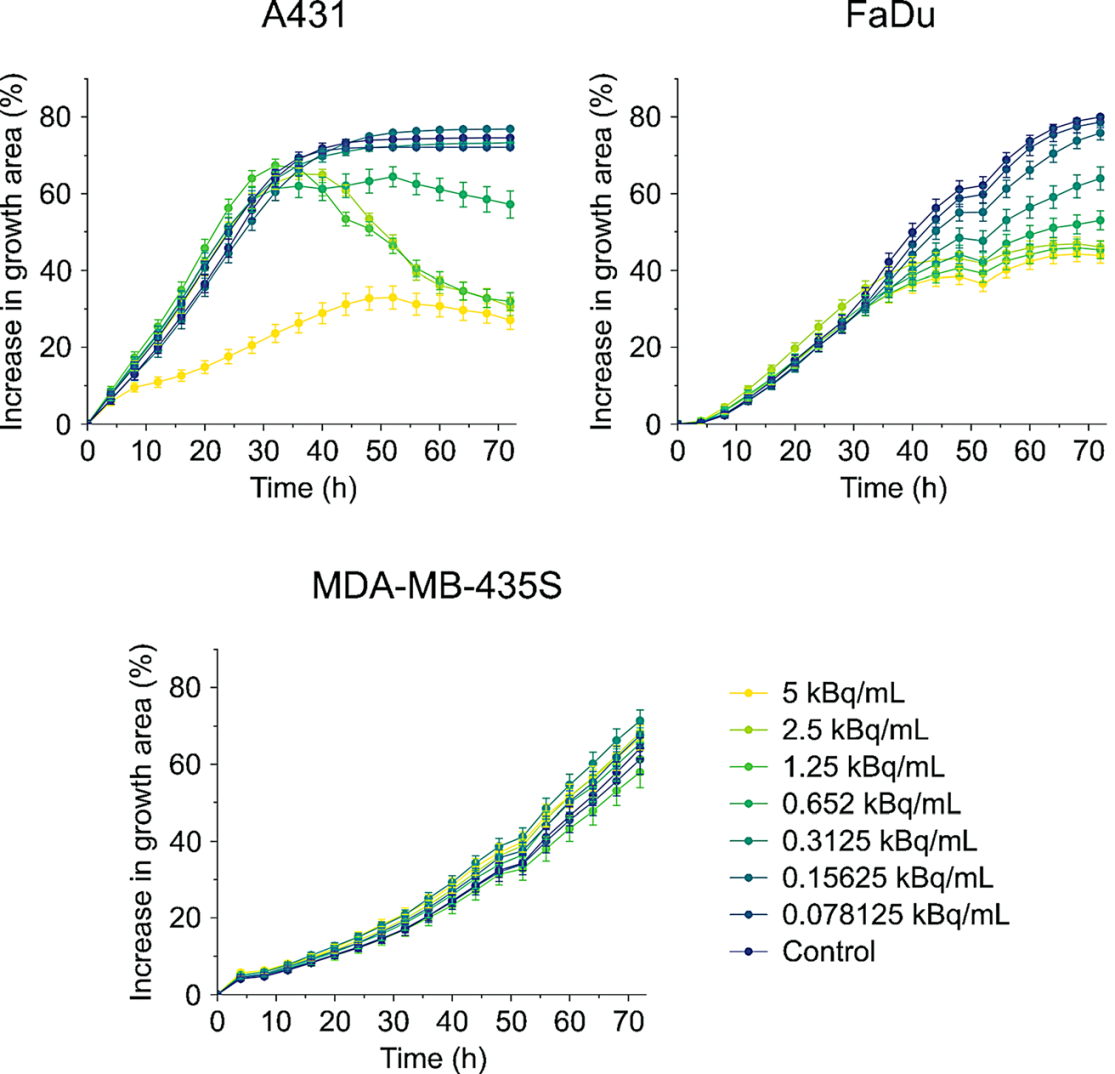
Antiproliferative effect of ^225^Ac-labeled single-domain antibodies over time is dependent on the number of EGFR molecules per cell. Proliferating tumor cells with high (A431), moderate (FaDu) and low (MDA-MB-435 S) EGFR surface expression levels were incubated for 4 h with increasing activity concentrations of biparatopic [^225^Ac]Ac-mcp-AB single-domain antibodies and subsequently supplemented with fresh medium. Cells were cultured at 37 °C and 5% CO_2_ for 72 h and microscopic phase contrast images were acquired every 4 h

To further investigate the therapeutic efficacy of the biparatopic construct depending on the EGFR expression level, cytotoxicity was assessed in real time using Incucyte live-cell imaging (Fig. 7). The growth inhibition of cancer cells after treatment with [^225^Ac]Ac-mcp-AB correlates with both the administered dose and the EGFR density in the cell types. The most severe antiproliferative effect was observed for A431 and FaDu cells, while growth of MDA-MB-435 S cells, expressing only very low levels of EGFR, was only marginally affected even at the highest activity concentration applied.

The obtained in vitro toxicity data clearly demonstrate the superiority of the biparatopic ^225^Ac-labeled immunoconjugate in terms of cell killing capacity and antiproliferative activity.

**Fig. 8:**
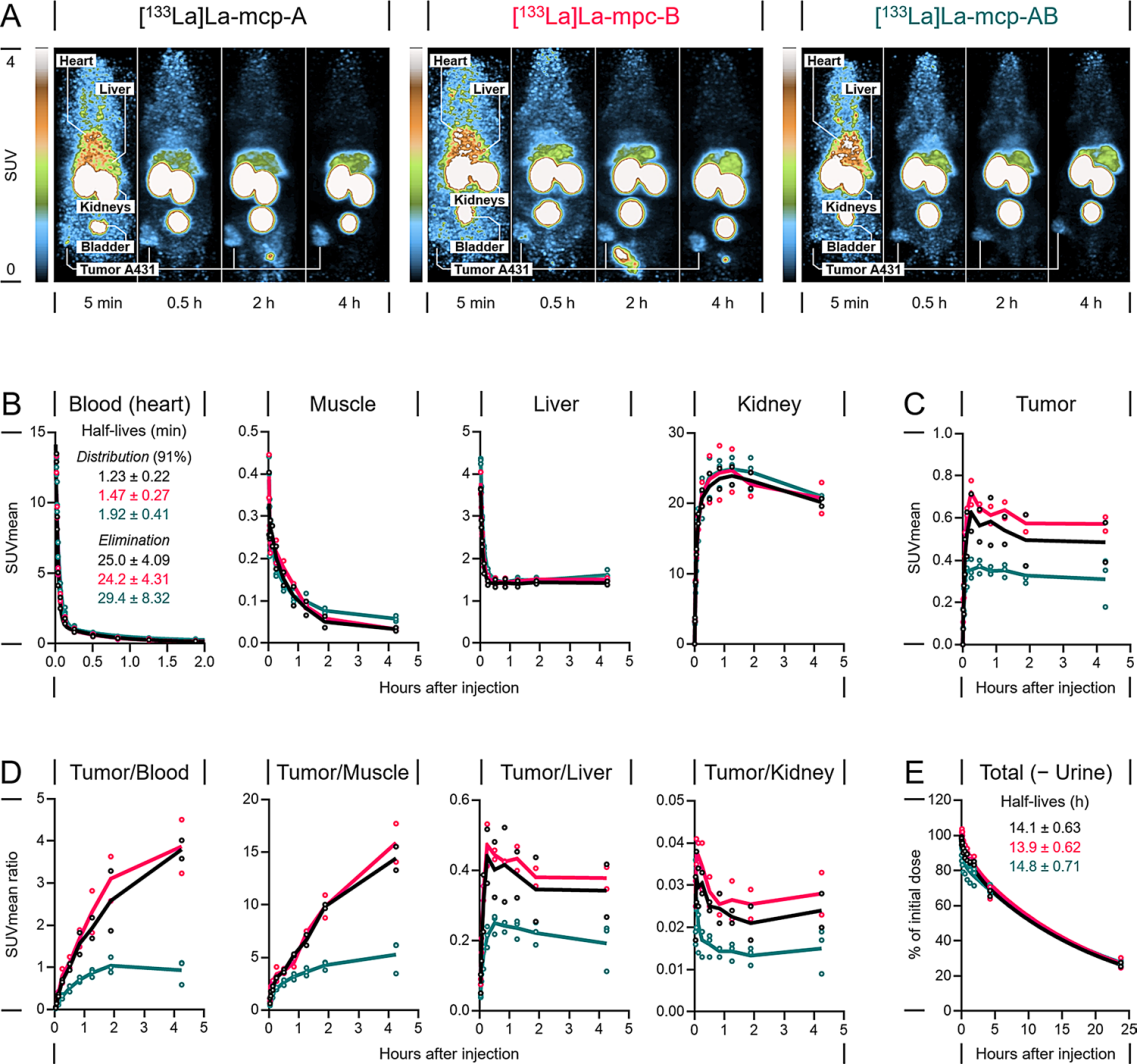
Distribution and tissue-specific pharmacokinetics of ^133^La-labeled immunoconjugates in A431 xenograft mice measured by PET imaging. (**A**) Maximum-intensity projections of PET images at indicated time points with common scale. (**B**-**C**) Kinetic profiles of activity concentrations in blood and indicated tissues. (**D**) Kinetic profiles of calculated tumor/tissue ratios. (**E**) Kinetic profiles of total body activity content

**Small-animal PET imaging using** ^**133**^**La-labeled single-domain antibodies.** The locoregional distribution of the two monovalent radioimmunoconjugates [^133^La]La-mcp-A and [^133^La]La-mcp-B and their biparatopic variant [^133^La]La-mcp-AB in a subcutaneous A431 tumor xenograft model was visualized via small-animal PET imaging up to 4 h after injection (Fig. 8A). Quantitative image analyses images provided kinetic profiles of region-averaged standardized uptake values (SUVmean) in blood (heart), liver, kidneys, muscle and tumors (Fig. 8B-C). All three radioimmunoconjugates showed biphasic blood kinetics with similar half-lives for distribution (1.23–1.92 min) and elimination (24.2–29.4 min) as well as similar uptake and retention in liver and kidneys (reaching 9.6 − 11% ID and 17 − 25% ID, respectively). In tumors, the two monovalent radioimmunoconjugates [^133^La]La-mcp-A and [^133^La]La-mcp-B showed similar uptake that was also higher compared to their biparatopic variant [^133^La]La-mcp-AB (Fig. 8C). All three radioimmunoconjugates reached the highest uptake in tumor tissue at time points < 30 min after injection followed by slow washout. Given their kinetic profiles in tumors, both [^133^La]La-mcp-A and [^133^La]La-mcp-B provided higher tumor contrast (tumor/tissue ratios) compared to [^133^La]La-mcp-AB (Fig. 8D). All three radioimmunoconjugates were eliminated from the animals with similar half-lives (13.9–14.8 h, Fig. 8E).

Since the in vitro and in vivo results showed largely similar characteristics of the two monovalent radioimmunoconjugates, the following ex vivo biodistribution data were collected exclusively for one of the two monovalent variants, in this case mcp-A, in comparison to the biparatopic mcp-AB, in order to reduce the number of experimental animals.

**Fig. 9:**
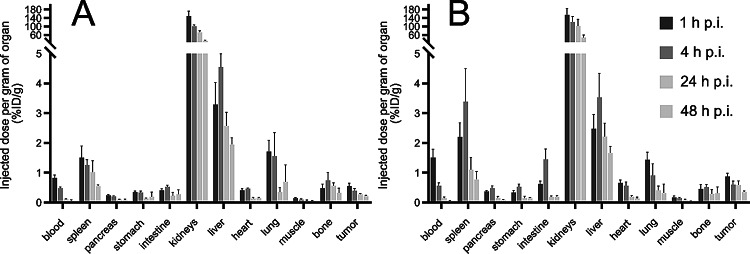
Ex vivo biodistribution of [^225^Ac]Ac-mcp-A (**A**) and [^225^Ac]Ac-mcp-AB (**B**) in mice bearing A431 tumor xenografts. The data is presented as the mean of the percentage of injected dose per gram of organ ± standard deviation (*n* = 5). The ordinate scale is modified in order to emphasize the uptake in all organs but kidneys

**Small animal biodistribution experiments using** ^**225**^**Ac-labeled immunoconjugates.** The organ distribution profile of [^225^Ac]Ac-mcp-A and [^225^Ac]Ac-mcp-AB was evaluated in subcutaneous A431 tumor-xenografted mice (Fig. 9, Supplemental Tables 1 and 2) upon injection of 50 kBq of each sdAb.

Their uptake in EGFR-expressing tumors peaked already 1 h p.i., whereby the accumulation was slightly higher for the biparatopic radioimmunoconjugate (0.87 ± 0.12% ID/g) compared to its monovalent counterpart (0.55 ± 0.10% ID/g). Although the amount of radioactivity in the tumor gradually decreases over time for both conjugates, this moderate difference remains evident at all time points. Apart from this, both ^225^Ac-labeled sdAbs show a substantial retention in the kidneys, which slowly decreases over the assessment period. However, the remaining amount of both radioimmunoconjugates in the kidneys is still comparatively high at 48 h p.i. with 32.24 ± 3.67% ID/g and 47.72 ± 11.80% ID/g for [^225^Ac]Ac-mcp-A and [^225^Ac]Ac-mcp-AB, respectively, which indicates a considerable unspecific retention in the renal cortex. Some retained radioactivity was also found in other organs such as the liver, spleen and lungs, but to a much lesser extent than in the kidneys. A closer look reveals that the biparatopic [^225^Ac]Ac-mcp-AB exhibits a higher uptake in the spleen in comparison to the monovalent variant [^225^Ac]Ac-mcp-A. Both radioimmunoconjugates also hardly differ from each other in terms of blood circulation time as the majority of the injected dose is cleared within the first 4 h after injection. Although the biparatopic construct has a slightly higher activity in the blood pool after 1 h p.i. than the monovalent one, the values tend to converge over the remaining examination period.

**Fig. 10:**
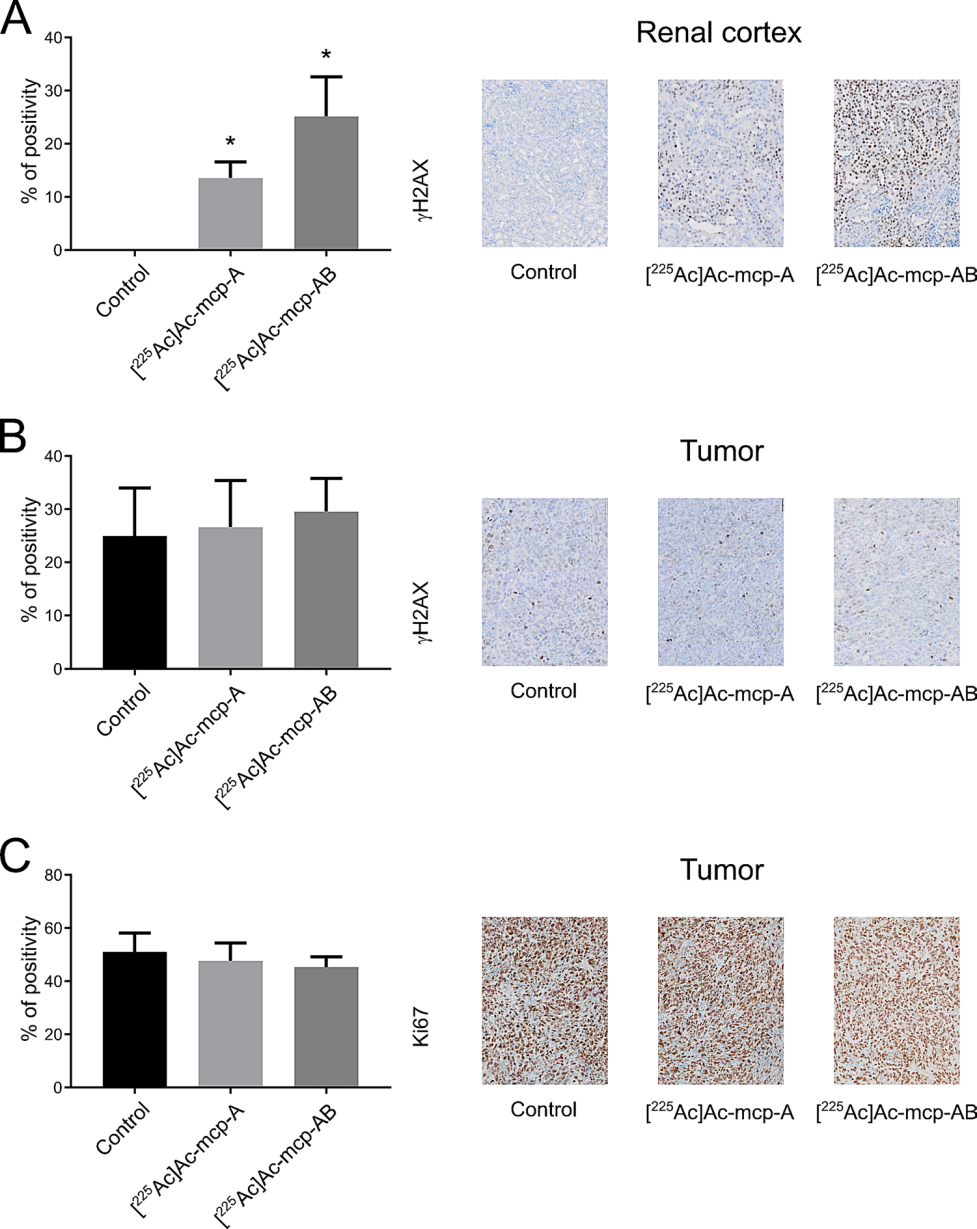
Histological assessment of organ sections originating from mice treated with 50 kBq of [^225^Ac]Ac-mcp-A and [^225^Ac]Ac-mcp-AB, respectively, in comparison to control animals. Immunohistochemical detection and quantification of DNA double-strand breaks in kidney (**A**) and tumor (**B**) sections by staining against γH2AX as well as indication of tumor cell proliferation (**C**) by anti-Ki67 staining. Values are expressed as mean with standard deviations. P-values *p* < 0.05 are indicated with * (*n* = 5)

**Histological examination of tumor and kidney tissues.** At 48 h after injection of 50 kBq of [^225^Ac]Ac-mcp-A and [^225^Ac]Ac-mcp-AB, respectively, tissue samples of tumors and kidneys were examined histologically for DNA damage (Fig. 10).

Immunostaining of kidney sections against phospho-H2AX (γ-H2AX) clearly indicated the presence of DNA double-strand breaks in renal cortex of ^225^Ac-radioimmunoconjugate-treated mice, with no evidence of DNA damage in kidneys of control mice (Fig. 10A). Noticeably more DNA double-strand breaks were detected in the kidney sections of those mice receiving the biparatopic conjugate. As a result, DNA damage could even be detected in the renal medulla of those mice injected with [^225^Ac]Ac-mcp-AB. However, this was not the case in mice that received [^225^Ac]Ac-mcp-A, where γ-H2AX positivity was limited to the cortex.

Histological analysis of the corresponding tumor tissues showed moderate DNA damage in all three treatment groups at 48 h p.i., including the control animals (Fig. 10B). However, no significant difference in the number of γ-H2AX-positive cells was observed between the respective experimental groups. This observation suggests that the detected DNA double-strand breaks originate from natural genomic instability of this subcutaneous tumor model and are not related to the injection of the ^225^Ac-labeled immunoconjugates.

The subsequent examination of tumor cell proliferation using immunohistochemical visualization of the corresponding marker Ki67 showed no significant differences between ^225^Ac-treated mice and untreated controls (Fig. 10C).

In conclusion, there was no obvious therapeutic effect of the ^225^Ac-labeled immunoconjugates on the tumor cells at these early time points, whereas the first indications of nephrotoxicity were observed with the applied dose of 50 kBq.

## Discussion

The increasing clinical implementation and related growing importance of ^225^Ac-based TAT necessitates appropriate diagnostic imaging surrogates for detailed radiopharmacological characterization, dose estimation and treatment monitoring. Due to its attractive physical properties such as low-energy positron emission and acceptable co-emission of low-intensity gamma rays, ^133^La has become one of the frontrunners as PET imaging counterpart for ^225^Ac. In combination with the macrocyclic chelator macropa allowing efficient radiolabeling at rt, ^133^La represents a genuine alternative to the short-lived PET nuclide ^68^Ga, in particular when it comes to the application of sensitive, antibody-based vector molecules.

In order to confirm this hypothesis, we initially engineered different sdAb fusion proteins and introduced a single macropa chelator at their respective C terminus, as this part is not involved in antigen binding. The obtained immunoconjugates could be effectively radiolabeled with both ^133^La (30 MBq/nmol) and ^225^Ac (0.5 MBq/nmol) within 15 min at rt without the need for post-labeling purification. For stability studies, the immunoconjugates were labeled with ^225^Ac and were found to be intact for more than 10 d in PBS as well as human serum.

The in vitro binding, internalization and co-localization studies with both the ^133^La-labeled and the CY3-labeled sdAbs showed similar binding behavior of the two monovalent sdAbs, while the biparatopic derivative is characterized by substantially increased cellular uptake. Such receptor-mediated internalization is preferentially observed with bivalent and biparatopic ligands, since bridging structures can be formed upon antigen binding. Intramolecular bridging occurs when the two antigen-binding domains bind simultaneously to the different epitopes of the identical receptor molecule. In contrast, the simultaneous interaction of bivalent or biparatopic ligands with the epitopes of different receptors leads to the formation of intermolecular crosslinks, inducing a variety of physiological effects including endocytosis, lysosomal antigen degradation and downregulation of associated cell signaling (Akiba and Tsumoto 2020). Antagonistic effects such as inhibition of EGF-dependent receptor phosphorylation, cell proliferation and tumor growth have been described for various EGFR-specific biparatopic molecules (Roovers et al. 2011; Heukers et al. 2013; Fan et al. 2021; Sharifi et al. 2021). Enhanced internalization into tumor cells is particularly beneficial for the effective targeted delivery of α-emitting radionuclide generators (^225^Ac, ^212^Pb) by increasing the likelihood for severe, irreparable DNA damage and by minimizing at the same time the release of the recoiling daughter nuclides (McDevitt et al. 2001). Therefore, the considerably enhanced EGFR-specific internalization of the biparatopic derivative is also the apparent reason for the substantially higher level of cytotoxicity as well as for the stronger antiproliferative effect of this particular ^225^Ac-labeled sdAb compared to its monovalent counterparts. Its inhibitory effect on cell growth and clonogenic activity is not only concentration-dependent, as expected, but also depends on the EGFR density of the analyzed cancer cell lines highlighting the in vitro target specificity of this sdAb-based TAT strategy.

Subsequent to this comprehensive in vitro evaluation, the generated immunoconjugates were further analyzed in detail with regard to their pharmacokinetics, biodistribution and tumoritropic accumulation. Small animal PET imaging using the ^133^La-labeled sdAbs revealed a comparable tissue profile with fast blood clearance and no substantial accumulation in healthy tissues apart from the kidneys and liver. The slightly higher tumor accumulation of the monovalent immunoconjugates could be due to their smaller molecule size, which is associated with faster and deeper diffusion into the tumor (Thurber et al. 2008; Schmidt and Wittrup 2009). In addition, the biparatopic nature of mcp-AB, leading to a higher avidity for the target structure, could be a reason for the lower tumor penetration. As with conventional bivalent antibodies, biparatopicity can result in efficient trapping in regions of first antigen recognition at the point of extravasation (Rudnick et al. 2011; Xenaki et al. 2017; Jumapili et al. 2023). This binding-site barrier effect is assumed to be responsible for antibody sequestration and a rather heterogeneous intratumoral distribution, which often comprises only a few cell layers around the adjacent blood vessels (Fujimori et al. 1990; Juweid et al. 1992; Weinstein and van Osdol 1992; Rudnick and Adams 2009).

A landmark study by Debbie et al. recently highlighted the clear advantage of monomeric sdAbs in terms of rapid and homogeneous tumor accumulation compared to dimeric constructs (Debie et al. 2020). The authors attribute this obvious inferiority on the one hand to the larger size of the dimeric sdAbs hindering extravasation and slowing diffusion, and on the other hand to the increased avidity of the bivalent formats, which leads to a restricted intratumoral distribution due to the binding-site barrier effect. The benefit of monomeric constructs in terms of tumor targeting capacity is also emphasized in a number of other relevant studies, underlining the effect of molecular size of the sdAb-based tracer on tumor uptake and highlighting the value of monomeric formats for homogenous tumor penetration (Movahedi et al. 2012; Krasniqi et al. 2018; Beltran Hernandez et al. 2019).

Labeled with ^225^Ac, the different sdAbs immunoconjugates were evaluated by ex vivo dissection analyses, largely confirming the outcomes of the prior PET imaging study, but with the biparatopic ^225^Ac-radioimmunoconjugate showing slightly higher tumor accumulation. Despite this slight deviation in tumor accumulation observed for the ^133^La-labeled radioimmunoconjugates and their therapeutic counterparts, the biodistribution of the monomeric and biparatopic variants is largely consistent. The massive accumulation and persistent retention in the kidneys resulted in severe DNA damage compared to untreated controls, which was demonstrated by immunohistochemical analysis of DNA double-strand breaks in corresponding tissue sections. In contrast, the insufficient tumor accumulation of both ^225^Ac-radioimmunoconjugates led to no significant difference in the DNA damage observed there, nor to significantly reduced tumor cell proliferation compared to the control group.

Based on the comprehensive biodistribution data obtained, the obvious conclusion can be drawn that a drastically reduced renal retention along with substantially increased tumor accumulation using a small-sized monovalent format with a high tumor penetrating potential is indispensable for sdAb-based TAT (Jumapili et al. 2023).

## Conclusions

This current study demonstrates, to the best of our knowledge, for the first time the efficient, straightforward and gentle radiolabeling of temperature-sensitive macropa-functionalized sdAbs with the α-emitter ^225^Ac and its PET imaging surrogate ^133^La while preserving the excellent binding properties for their molecular target EGFR. Using EGFR-directed, monovalent and biparatopic sdAb formats as examples, we performed an intensive preclinical characterization and compared the different derivatives in vitro and in vivo with regard to their cell binding, internalization and cytotoxicity as well as their pharmacokinetics, biodistribution and tumor accumulation. The obtained results presented herein clearly indicate that ^133^La is an excellent radiometal for high-resolution PET imaging and thus forms a valuable theranostic radionuclide pair in combination with ^225^Ac.

## Materials and methods

### Synthesis of **BCN-PEG**_**5**_**-mcp**

All chemicals were purchased from commercial suppliers and used without further purification. TLC analyses for reaction control were performed on Merck Silica Gel 60 F_254_ TLC plates and visualized using 254 nm UV light. Analytical HPLC was performed on VWR Hitachi (L2000 series) using analytical Zorbax 300SB-C18 column, 100 × 4.6 mm (Agilent Technologies, Waldbronn, Germany) and acetonitrile/water (0.1% TFA each) as mobile phase using a flow rate of 1 mL/min. Chromatographic separations were performed using automated flash column chromatography on Isolera Four (Biotage, Uppsala, Sweden) using silica gel cartridges (SNAP HC-Sfär; 5 g, 10 g, or 25 g) and HPLC system Knauer Azura (Knauer, Berlin, Germany) with Zorbax 300SB-C18 semi-preparative column (Agilent Technologies, Waldbronn, Germany) using acetonitrile/water (0.1% TFA each) as mobile phase with a flow rate of 6 mL/min. Compound **6** was synthesized according to the literature (Reissig et al. 2021). NMR spectra were recorded at 25 °C using an Agilent DD2 400 MHz NMR or Agilent DD2 600 MHz NMR spectrometer with ProbeOne. Chemical shifts are expressed in parts per million (ppm) and coupling constants *J* in Hz. ^1^H and ^13^C spectra were internally referenced using the signal of the deuterated solvent. Mass spectra were recorded on an Advion ExpressIon compact mass spectrometer using electrospray ionization (ESI).

**Dimethyl 4-((14-azido-3**,**6**,**9**,**12-tetraoxatetradecyl)oxy)pyridine-2**,**6-dicarboxylate (3).** 14-Azido-3,6,9,12-tetraoxatetradecyl tosylate (**1**, 420 mg, 1.01 mmol), dimethyl 4-hydroxypyridine-2,6-dicarboxylate (**2**, 319 mg, 1.51 mmol), and K_2_CO_3_ (529 mg, 3.83 mmol) were dissolved in anhydrous acetonitrile (10 mL) and stirred at 85 °C overnight. After cooling to rt, the solids were filtered, the solvent was removed and the crude product was purified via automated column chromatography (chloroform: ethyl acetate = 10:1) to yield compound **3** (190 mg, 41%) as colorless syrup. ^1^H NMR (400 MHz, CDCl_3_): δ = 3.38 (t, ^3^*J* = 5.1 Hz, 2 H, CH_2_), 3.64–3.69 (m, 12 H, OCH_2_), 3.70–3.74 (m, 2 H, OCH_2_), 3.90 (t, ^3^*J* = 4.6 Hz, 2 H, CH_2_), 4.00 (s, 6 H, CH_3_), 4.31 (t, ^3^*J* = 4.6 Hz, 2 H, CH_2_), 7.83 (s, 2 H, Ar-H) ppm; ^13^C NMR (101 MHz, CDCl_3_): δ = 50.8 (CH_2_), 53.4 (CH_3_), 68.6, 69.2, 70.2, 70.7, 70.8, 70.8, 70.9, 71.1 (8 x CH_2_), 114.8 (CH_Ar_), 149.9 (C_Ar_), 165.3 (C_Ar_), 167.1 (C = O) ppm. MS (ESI+): *m/z* = 457 [M + H]^+^, 479 [M + Na]^+^.

**Methyl 4-((14-azido-3**,**6**,**9**,**12-tetraoxatetradecyl)oxy)-6-(hydroxymethyl)picolinate (4).** Compound **3** (385 mg, 0.84 mmol) was dissolved in anhydrous methanol (10 mL) and NaBH_4_ (32 mg, 0.84 mmol) was added. The mixture was stirred for 5 h at 65 °C. Afterwards, the solvent was removed, saturated NH_4_Cl solution was added, the aqueous phase extracted with chloroform (3 × 10 mL). The combined organic phases were dried over Na_2_SO_4_, the solvent was removed and purification was done via automated column chromatography (chloroform: ethyl acetate = 10:1) to yield compound **4** (190 mg, 53%) as colorless syrup. ^1^H NMR (400 MHz, CDCl_3_): δ = 3.37 (t, ^3^*J* = 5.0 Hz, 2 H, CH_2_), 3.62–3.68 (m, 12 H, OCH_2_), 3.68–3.71 (m, 2 H, OCH_2_), 3.87 (t, ^3^*J* = 4.7 Hz, 2 H, CH_2_), 3.96 (s, 3H, CH_3_), 4.24 (t, ^3^*J* = 4.6 Hz, 2 H, CH_2_), 4.78 (s, 3H, CH_3_), 7.09 (d, ^4^*J* = 2.3 Hz, 1H, Ar-H), 7.56 (d, ^4^*J* = 2.3 Hz, 1H, Ar-H) ppm; ^13^C NMR (101 MHz, CDCl_3_): δ = 50.8 (CH_2_), 53.1 (CH_3_), 64.8, 68.1, 69.4, 70.1, 70.7, 70.7, 70.8, 70.8, 70.9, 71.1 (10 x CH_2_), 109.8 (CH_Ar_), 111.3 (CH_Ar_), 148.6 (C_Ar_), 162.4 (C = O), 165.6 (C_Ar_), 166.8 (C_Ar_) ppm. MS (ESI+): *m/z* = 429 [M + H]^+^, 451 [M + Na]^+^.

**Methyl 4-((14-azido-3**,**6**,**9**,**12-tetraoxatetradecyl)oxy)-6-(chloromethyl)picolinate (5).** Compound **4** (140 mg, 0.33 mmol) was cooled to 0 °C, SOCl_2_ (1 mL) was slowly added and the mixture was stirred at 0 °C for 1 h and at rt for 2 h. Afterwards, the mixture was added dropwise to an ice-cold saturated hydrogen carbonate solution (15 mL). The aqueous solution was extracted with dichloromethane (3 × 15 mL) to yield compound **5** (120 mg, 82%) as a colorless syrup without further purification. ^1^H NMR (400 MHz, CDCl_3_): δ = 3.37 (t, ^3^*J* = 4.5 Hz, 2 H, CH_2_), 3.62–3.68 (m, 12 H, OCH_2_), 3.69–3.74 (m, 2 H, OCH_2_), 3.89 (t, ^3^*J* = 4.2 Hz, 2 H, CH_2_), 3.99 (s, 3H, CH_3_), 4.26 (t, ^3^*J* = 4.1 Hz, 2 H, CH_2_), 4.71 (s, 3H, CH_3_), 7.25 (br s, 1H, Ar-H), 7.62 (br s, 1H, Ar-H) ppm; ^13^C NMR (101 MHz, CDCl_3_): δ = 46.4 (CH_2_), 50.8 (CH_2_), 53.1 (CH_3_), 68.2, 69.3, 70.2, 70.7, 70.8, 70.8, 70.8, 71.1 (8 x CH_2_), 111.5 (CH_Ar_), 112.6 (CH_Ar_), 149.2 (C_Ar_), 158.9 (C = O), 165.5 (C_Ar_), 166.9 (C_Ar_) ppm. MS (ESI+): *m/z* = 447 [M + H; ^35^Cl]^+^, 449 [M + H; ^37^Cl]^+^, 469 [M + Na; ^35^Cl]^+^, 471 [M + H; ^37^Cl]^+^.

**Methyl 6-((16-(3-((14-azido-3**,**6**,**9**,**12-tetraoxatetradecyl)oxy)-5-(methoxycarbonyl)benzyl)- 1**,**4**,**10**,**13-tetraoxa-7**,**16-diazacyclooctadecan-7-yl)methyl)picolinate (7).** Compound **5** (105 mg, 0.23 mmol), methyl 6-((1,4,10,13-tetraoxa-7,16-diazacyclooctadecan-7-yl)methyl)-picolinate (**6**, 194 mg, 0.47 mmol), diisopropylethylamine (0.5 mL), and NaI (approx. 10 mg) were dissolved in anhydrous acetonitrile (10 mL) and the mixture was stirred at 85 °C overnight. After TLC control, the solvent was removed and the crude product was purified via automated column chromatography (ethyl acetate: ethanol = 1:0 → 0:1) to yield compound **7** (192 mg, 99%) as colorless syrup. ^1^H NMR (400 MHz, CDCl_3_): δ = 3.38 (t, ^3^*J* = 5.0 Hz, 2 H, CH_2_), 3.61–3.73 (m, 34 H, OCH_2_), 3.85–4.00 (m, 18 H, 2 x CH_3_ + OCH_2_), 4.27 (t, ^3^*J* = 3.9 Hz, 2 H, CH_2_), 4.68 (s, 3H, CH_3_), 4.78 (s, 3H, CH_3_), 7.34 (br s, 1H, Ar-H), 7.65 (br s, 1H, Ar-H), 7.76 (d, ^3^*J* = 7.7 Hz, 1H, Ar-H), 7.96 (t, ^3^*J* = 7.7 Hz, 1H, Ar-H), 8.12 (d, ^3^*J* = 7.7 Hz, 1H, Ar-H) ppm; ^13^C NMR (101 MHz, CDCl_3_): δ = 50.8 (CH_2_), 53.2 (CH_3_), 54.6, 57.1, 57.3, 65.1, 65.2, 68.2, 69.2, 70.1, 70.4, 70.5, 70.6, 70.7, 70.7, 70.9 (8 x CH_2_), 113.4 (CH_Ar_), 113.9 (CH_Ar_), 125.6 (CH_Ar_), 128.4 (CH_Ar_), 139.2 (CH_Ar_), 147.8 (C_Ar_), 149.0 (C_Ar_), 150.6 (C = O), 152.0 (C = O), 164.8 (C_Ar_), 167.4 (C_Ar_) ppm. MS (ESI+): *m/z* = 411 [M + 2 H]^2+^, 822 [M + H]^+^, 860 [M + K]^+^.

**6-((16-(3-((1-(Bicyclo[6.1.0]non-4-yn-9-yl)-3-oxo-2**,**7**,**10**,**13**,**16-pentaoxa-4-azaoctadecan-18-yl)oxy)-5-carboxybenzyl)-1**,**4**,**10**,**13-tetraoxa-7**,**16-diazacyclooctadecan-7-yl)methyl)picolinic acid (BCN-PEG**_**5**_**-mcp).** Compound **7** (90 mg, 0.11 mmol) was dissolved in methanol (5 mL) and Pd/C (20 mg) was added. The flask was flushed with H_2_ and stirred vigorously at rt overnight under H_2_ atmosphere. After HPLC control, the mixture was filtered, the solvent was removed and the yielded product was used without purification (80 mg, 92%) as colorless syrup. Afterwards, the amine (80 mg, 0.10 mmol) was dissolved in a mixture of methanol and water (3 mL, v:v = 1:1), LiOH (14 mg) was added and the mixture was stirred at rt for 3 h. After HPLC control, the solvent was removed and the crude product was purified via semipreparative HPLC (gradient: 10% → 30% ACN in water + 0.1% TFA) to give the deprotected compound (35 mg, 45%) as colorless syrup after lyophilization. Finally, the deprotected compound (35 mg, 0.05 mmol), exo-BCN-pNPE (16 mg, 0.05 mmol), and Et_3_N (20 µL) were dissolved in anhydrous acetonitrile (2 mL) and stirred at rt overnight. Afterwards, the solvent was removed and crude product was purified via semipreparative HPLC (gradient: 10% → 30% ACN in water + 0.1% TFA) to give **BCN-PEG**_**5**_**-mcp** (15 mg, 35%; 15% over 3 steps) as colorless syrup after lyophilization. ^1^H NMR (400 MHz, CD_3_CN): δ = 0.57–0.74 (m, 3H, BCN), 1.26–1.40 (m, 2H, BCN), 2.08 (br. d, ^2^*J* = 15.8 Hz, 2H, BCN), 2.17–2.37 (m, 4 H, BCN), 3.21 (q, ^3^*J* = 5.6 Hz, CH_2_), 3.41–3.66 (m, 30 H, CH_3_ + CH_2_), 3.79–3.93 (m, 12 H, CH_2_), 4.31 (t, ^3^*J* = 4.2 Hz, 3H, CH_2_), 4.64 (br. s, 2H, CH_2_Ar), 4.78 (br. s, 2H, CH_2_Ar), 5.62 (br. s, 1H, NH), 7.20 (s, 1H, Ar-H), 7.62–7.69 (m, 2H, Ar-H), 8.05 (t, ^3^*J* = 7.7 Hz, 1H, Ar-H), 8.13 (d, ^3^*J* = 7.7 Hz, Ar-H) ppm; ^13^C NMR (101 MHz, CDCl_3_): δ = 21.8, 23.6, 24.7, 34.1 (4 x BCN), 41.5 (CH_2_), 55.2 (BCN), 58.1, 58.5, 66.2, 66.3, 69.3, 69.7, 69.8, 70.5, 70.8, 70.9, 71.1, 71.2, 71.4 (CH_2_), 99.7 (C ≡ C), 111.2, 114.1, 124.9, 128.0, 140.6 (5 x CH_Ar_), 148.2, 149.9, 157.7 (C_Ar_), 165.7 (C = O), 166.2 (C = O), 168.9 (C = O_BCN_) ppm. MS (ESI+): *m/z* = 944 [M + H]^+^, 966 [M + Na]^+^.

### Construction, expression and purification of recombinant single-domain antibodies

Cloning of the monovalent and biparatopic anti-EGFR single domain antibodies into the bacterial expression vector pET-28b, their cytoplasmic expression in *Escherichia (E.) coli* SHuffle T7 Express as well as their purification by immobilized metal affinity chromatography (IMAC) were described in detail previously (Singh et al. 2020). The monovalent anti-EGFR single domain antibodies A and B are based on camelid VHH domains derived from the clone 7C12 and 9G8 respectively, described in the literature (Gainkam et al. 2008; Roovers et al. 2011; Schmitz et al. 2013). Their biparatopic form was constructed as genetic fusion with the monovalent domains connected by a flexible (GGGGS)_3_ linker. The corresponding synthesized coding sequences were purchased from the company Eurofins Genomics with 5′ *Nco*I and 3′ *Hin*dIII restriction enzyme sites to allow insertion into the vector pET-28b: Strep-sortag-6HIS. This plasmid was derived from the commercially available pET-28b vector (Merck) by addition of a DNA fragment coding for a (GGGGS)_3_ spacer followed by a Strep-tag, the LPETGG sortase motif and another (GGGGS)_3_ spacer (Singh et al. 2020). Following ligation into pET-28b: Strep-sortag-6HIS, DNA was transformed into chemically competent *E. coli* NEB 5-alpha cells (New England Biolabs) and the sequence of the recombinant constructs was verified by Sanger sequencing (Eurofins Genomics). To allow for cytoplasmic expression of proteins, the vectors were transformed into *E. coli* SHuffle T7 Express (New England Biolabs) and MagicMedia E. coli Expression Medium (Life Technologies) was used as described recently (Singh et al. 2020). After expression, the hexahistidine tagged single-domain antibodies were purified by IMAC using a high-capacity Ni-iminodiacetic acid resin (Clontech Laboratories) in combination with an ÄKTA pure chromatography system (GE Healthcare) (Karges et al. 2020).

### Site-specific modification of recombinant single-domain antibodies

The two-step site-specific antibody modification approach using a combination of Sortase A-mediated bioconjugation and SPAAC was described recently in detail (Singh et al. 2020). Briefly, equimolar amounts of hexahistidine tagged Sortase A and single-domain antibodies were incubated with a tenfold molar excess of (Gly)_3_-Lys-N_3_ (Iris Biotech GmbH) in sortase buffer (50 mM Tris-HCl, 150 mM NaCl and 10 mM CaCl_2_, pH 7.5) at 30 °C for 4 h with gentle shaking. All remaining hexahistidine tagged proteins were removed from the reaction mixture by IMAC using prepacked His60 Ni Gravity Columns (Clontech Laboratories). The unreacted (Gly)_3_-Lys-N_3_ was removed by affinity chromatography using a Strep-Tactin XT system (IBA Lifesciences) in combination with an ÄKTA pure chromatography system (GE Healthcare). The resulting azide-functionalized single-domain antibodies were reacted with a twentyfold molar excess of BCN-PEG_5_-mcp or a tenfold molar excess of dibenzylcyclooctyne-Sulfo-Cy3 (DBCO-Sulfo-Cy3, Jena Bioscience GmbH), respectively, at 25 °C for 4 h with gentle shaking. Non-conjugated BCN-PEG_5_-mcp and DBCO-Sulfo-Cy3 were removed by size-exclusion chromatography using Zeba Spin Desalting Columns (7 K, Thermo Scientific) with elution in 0.2 M ammonium acetate buffer (pH 6) and subsequent spin filtration with Amicon Ultra-0.5 centrifugal filter devices with a molecular weight cutoff (MWCO) of 3 K (Amicon Ultra 3 kDa cutoff, Merck).

### Radiolabeling, quality control and stability of macropa conjugates

[^133^La]LaCl_3_ was produced at the Helmholtz-Zentrum Dresden-Rossendorf with the 30 MeV TR-Flex-Cyclotron (Advanced Cyclotron Systems) using the ^134^Ba(p,2n)^133^La nuclear reaction as reported previously (Brühlmann et al. 2022, 2024). ^133^La-labeling was performed using ~ 30 MBq of [^133^La]LaCl_3_ per nmol of the respective macropa-functionalized single-domain antibody.

[^225^Ac]AcCl_3_ was purchased from ITM (Isotope Technologies Munich SE) and radiolabeling was performed using ~ 500 kBq of [^225^Ac]AcCl_3_ per nmol of the respective macropa-functionalized single-domain antibody.

The labeling reactions were set up in 0.2 M ammonium acetate buffer (pH 6) and incubated at rt for 15 min with gentle shaking. The extent of radiometal complexation was assessed by radio-TLC as described recently (Reissig et al. 2021, 2022). The normal phase system uses silica gel TLC plates as stationary phase and 50 mM EDTA solution (pH 7) as mobile phase, whereas TLC plates with RP-18 modified silica gel in acetonitrile/water (7/3) are used for the reverse phase system. The TLC plates were imaged using an Amersham Typhoon 5 Scanner (Cytiva Europe GmbH). For the radio-TLC analysis of the ^225^Ac-labeled immunoconjugates, a minimum decay time of 4 h was allowed before imaging the TLC plates to ensure that initial decay products did not interfere and only ^225^Ac-related signals are displayed. In addition, ^133^La-labeled immunoconjugates were analyzed by sodium dodecyl sulfate-polyacrylamide gel electrophoresis (SDS-PAGE) under non-reducing conditions. Briefly, 100 pmol of each radioimmunoconjugate in 5 µL of 0.2 M ammonium acetate buffer (pH 6) were mixed with 5 µL of 2x Laemmli sample buffer (Bio-Rad Laboratories) and incubated at 95 °C for 5 min. The samples were cooled to rt and added to the wells of a 15% SDS-polyacrylamide gel. The discontinuous SDS-PAGE was run at rt and 80 V until the dye front reached the resolving gel and then increased up to 160 V. After electrophoresis, the gel was imaged using an Amersham Typhoon 5 Scanner (Cytiva Europe GmbH) and stained with PageBlue protein staining solution (Thermo Fisher Scientific) according to the manufacturer’s instructions.

Stability of ^225^Ac-labeled immunoconjugates was investigated by incubation in phosphate-buffered saline (PBS) at 25 °C or in human serum (HS) at 37 °C, respectively. Aliquots (1 µL) were taken over a period of up to 10 d and analyzed using radio-TLC (normal phase system).

### Cell culture

The epidermoid carcinoma cell line A431 (ATCC^®^ Number: CRL-1555), the squamous cell carcinoma cell line FaDu (ATCC^®^ Number: HTB-43) as well as the melanoma cell line MDA-MB-435 S (ATCC^®^ Number: HTB-129) were purchased from American Type Culture Collection and cultured as previously reported (Zarschler et al. 2013, 2014). All cell lines were confirmed to be mycoplasma-negative using the Venor GeM Advance Mycoplasma Detection Kit (Minerva Biolabs) by monthly testing.

### Enzyme-linked immunosorbent assay (ELISA) for quantification of full-length EGFR in human cell lysates

The assays were conducted in accordance with the manufacturer’s protocol (Thermo Fisher Scientific, Invitrogen KHR9061). In brief, 10^8^ cells from A431, FaDu, and MDA-MB-435 S lines were washed twice with cold PBS at 4 °C and lysed in 1 mL of extraction buffer (containing 1 mM PMSF and 50 µL protease inhibitor cocktail) for 30 min on ice, with vortexing every 10 min. Post-centrifugation at 13,000 rpm for 10 min at 4 °C, the supernatant was transferred to clean tubes for protein quantification (Bio-Rad Laboratories), enabling appropriate dilutions. 100 µL of HuEGFR standards (0 to 20 ng/mL) were pipetted into the ELISA wells (96-well microplate), parallel with 100 µL of cell lysates. Following a 2-hour incubation at rt and four washes, the samples were incubated with huEGFR detection antibody solution for 1 h at rt, followed by four washes. Subsequently, 100 µL of anti-rabbit IgG HRP solution was added, followed by four additional washes. Samples were then incubated with 100 µL of stabilized chromogen for 30 min in the dark, after which 100 µL of stop solution was added, resulting in a color change from blue to yellow. Absorbance was measured at 450 nm.

### Cell binding and uptake studies

Saturation binding studies to determine the *K*_d_ and B_max_ were performed as described elsewhere (Reissig et al. 2021). In brief, 50,000 A431 or FaDu cells were seeded in 48-well microplates in 250 mL DMEM (Thermo Fisher Scientific) supplemented with 10% fetal calf serum (FCS, Merck) and cultivated for 48 h to allow cell adhesion and growth. During the experiment, cells were kept on ice and all reagents were added ice-cold. The cell culture medium was replaced by 150 µL of 1% bovine serum albumin (BSA, AppliChem) in Dulbecco’s phosphate-buffered saline (DPBS, Thermo Fisher Scientific) to assess total binding or 150 µL of 1% BSA/DPBS and 10 µM of unlabeled single-domain antibodies to determine nonspecific binding, respectively. The ^133^La-labeled single-domain antibodies were step-wise diluted to eight different concentrations ranging from 1.5625 nM to 200 nM in 1% BSA/DPBS. Then, 150 µL of these dilutions were added to the wells resulting in final radioligand concentrations of 0.78125 nM to 100 nM, and the cell culture microplates were further incubated for 90 min at 4 °C. After the incubation time, the cells were washed three times with ice-cold DPBS. Finally, cell lysis was achieved by the addition of 1% SDS/0.1 M NaOH and incubation for 30 min at rt with vigorous shaking. To quantify cell-associated radioactivity, an automatic gamma counter (Hidex Deutschland Vertrieb GmbH) was used. Total protein concentration in cell extracts was determined using the DC Protein Assay (Bio-Rad Laboratories) according to the manufacture’s microplate assay protocol using bovine serum albumin as protein standard. The *K*_d_ and B_max_ values were determined from the measured data using the Prism software (Version 10.1.2, GraphPad).

Internalization studies to measure time-dependent EGFR-mediated uptake into cells were performed according to a published procedure with slight modifications (Brandt et al. 2022; Cieslik et al. 2022). A total of 80,000 A431 or 70,000 FaDu cells were seeded in 48-well microplates in 250 mL DMEM supplemented with 10% FCS and cultivated overnight. The cell culture medium was replaced by 150 µL of 1% BSA/DPBS (total uptake) or 150 µL of 1% BSA/DPBS and 1 µM of unlabeled single-domain antibodies (nonspecific uptake). The ^133^La-labeled single-domain antibodies were diluted to 20 nM stock solutions using 1% BSA/DPBS. Then, 150 µL of these stock solutions were added to the wells resulting in final radioligand concentration of 10 nM, and the microplates were further incubated for 15 min, 30 min, 60 min, 120 min and 240 min at 37 °C and 4 °C, respectively. After incubation, the cells were washed with ice-cold DPBS. Cell surface-bound radioconjugates were removed by acid wash using ice-cold 50 mM glycine buffer (pH 2.8) twice for 5 min. After washing the cells once more with ice-cold DPBS, cell lysis was achieved by the addition of 1% SDS/0.1 M NaOH and incubation for 30 min at rt with vigorous shaking. Radioactivity and protein content of collected fractions was measured using an automatic gamma counter (Hidex Deutschland Vertrieb GmbH) and the DC Protein Assay (Bio-Rad Laboratories) according to the manufacture’s microplate assay protocol using bovine serum albumin as protein standard, respectively.

### Confocal microscopy

Confocal immunofluorescent analysis of A431 and FaDu cells was performed as published before with few modifications (Karges et al. 2020; Singh et al. 2020). Briefly, 50,000 cells per well were seated in an 8-chamber slide and cultivated overnight. The Cy3-conjugated sdAbs, A-Cy3, B-Cy3 or AB-Cy3, diluted in culture media was added to the wells at a concentration of 1 µM. After incubation times of 5 min, 15 min, 30 min, 1 h, 2 h and 4 h, the sdAb binding was stopped by removing the media, washing twice with ice-cold DPBS and subsequent fixation of the cells with 4% PFA/2.5% saccharose in DPBS. Cells were incubated with the fixing solution at 4 °C overnight and then washed three times with DPBS. Following this, cells were permeabilized with DPBS supplemented with 0.25% TritonX-100 at rt for 15 min and blocking was performed by incubation with 5% FCS in DPBS for 1 h at rt. Afterwards, cells were incubated with the fluorescent-labeled, anti-EGFR Alexa Fluor 647 antibody conjugate (Cell Signaling Technology, Inc.) diluted 1:300 in DPBS supplemented with 1% BSA and 0.25% TritonX-100, overnight at 4 °C. After antibody incubation, the cells were washed three times with DPBS before mounting the samples with ProLong^®^ Gold Antifade Reagent with DAPI (Cell Signaling Technology, Inc.) for 24 h at rt. Microscopy was performed with a Fluoview 1000 confocal laser scanning microscope (Olympus) using a 100x oil objective.

### Clonogenic assay

Colony formation of EGFR-expressing cells upon exposure to increasing activity concentrations of ^225^Ac-labeled single-domain antibodies was analyzed as previously reported with slight modifications (Cieslik et al. 2022; Reissig et al. 2022). A431 and FaDu cells were seeded in 12-well plates at a density of 100 cells per well containing 1 mL of DMEM with 10% FCS and cultivated overnight to allow cell adhesion. The cell culture medium was replaced by 1 mL of DMEM with 10% FCS containing ^225^Ac-labeled single-domain antibodies with elven different activity concentrations of 0.0025, 0.005, 0.01, 0.025, 0.05, 0.1, 0.25, 0.5, 1, 2.5 and 5 kBq/mL. After 4 h of incubation at 37 °C, the cell culture medium was removed and 1 mL of fresh DMEM with 10% FCS was added to each well. Plates were then incubated at 37 °C for seven (A431) and eleven (FaDu) days, respectively, and medium was renewed every three to four days. Finally, the culture medium was removed and colonies were stained with 1 mL of 0.5% crystal violet in 50% methanol. After 30 min, plates were rinsed three times with deionized water and air-dried. Plates were scanned with an Amersham Typhoon 5 Scanner (Cytiva Europe GmbH, Freiburg, Germany) and the colonies were counted using the Image-Quant TL software (Version 8.1, Cytiva Europe GmbH, Freiburg, Germany).

### Incucyte-based live-cell imaging and analysis

A431, FaDu and MDA-MB-435 S cells were seeded in 96-well plates with 3,000, 4,000 and 6,000 cells per well, respectively. After overnight cultivation, medium was removed and the ^225^Ac-labeled sdAb dilutions (5, 2.5, 1.25, 0.625, 0.3125, 0.15625 and 0.078125 kBq/mL), prepared in culture medium, were applied to the cells. After an incubation of 4 h at 37 °C, the medium containing ^225^Ac-labeled sdAbs was removed and the cells were washed with DPBS three times. Fresh medium was added to the cells and they were cultivated over 72 h at 37 °C in the Incucyte SX5 (Sartorius). Three phase-contrast pictures of each well were taken every 4 h. The confluence of each well was determined with the basic analysis tool using the AI-confluence analysis of the Incucyte software 2022 B Rev.

### PET imaging studies

PET imaging experiments with [^133^La]La-mcp-A, [^133^La]La-mcp-B, and [^133^La]La-mcp-AB experiments in A431 tumor-bearing mice were carried out according to the guidelines of the German Regulations for Animal Welfare. The protocols were approved by the local Ethical Committee for Animal Experiments (license 25-5131/562/52). Nude mice (Rj: NMIR-Foxn1^nu/nu^, female, 8 − 12 weeks old, Janvier Labs, Saint Berthevin Cedex, France) received a subcutaneous injection of 2 × 10^6^ A431 cells (ATCC, Manassas, VA, USA) into the right hind leg. Growth of A431 tumor xenografts was monitored by caliper measurements. When the tumor volume reached 200–300 mm^3^ (i.e., approx. 3 weeks after xenografting), mice were enrolled in the imaging study.

Anesthesia was induced and maintained with inhalation of 10% (v/v) desflurane in 30/10% (v/v) oxygen/air. During anesthesia, animals were continuously warmed at 37 °C. Each animal received 6 − 10 MBq (0.46 − 0.77 nmol) of the ^133^La-labeled radioimmunoconjugates delivered in Dulbecco’s phosphate-buffered saline via intravenous injection through a lateral tail vein catheter within the initial 30 s after scan start. Small-animal PET imaging was performed using the nanoScan^®^ PET/CT scanner (Mediso Medical Imaging Systems, Budapest, Hungary). Emission of the 511 keV annihilation photons was continuously recorded at a coincidence mode of 1 − 5. Two scans were performed within the time windows 0–2 h and 3.5–4.5 h after injection of the radioimmunoconjugates. With each scan, a corresponding CT image was recorded and used for anatomical referencing and attenuation correction. Animals were sacrificed 22 h after injection using CO_2_ inhalation and cervical dislocation and scanned for another three hours to determine the amount of activity remaining in the body. Binning, framing, and image reconstruction were performed as reported previously (Ullrich et al. 2016).

PET images were post-processed and analyzed using Rover version 3.0.77 h (ABX GmbH, Radeberg, Germany) and displayed as maximum intensity projections at indicated time points and common scaling. For extraction of region-averaged standardized uptake values (SUVmean), regions of interest (ROIs) were generated within spherical preselection masks in images (including all time frames) using tissue-specific threshold-based delineation (% of the image’s maximum voxel intensity) as follows: heart (> 15%, 0.1 cm^3^ of blood content), muscle (0%, 0.13 cm^3^), liver (> 60%, 0.2 cm^3^ of the right lobe); kidneys (> 60%, 0.2 cm^3^ of cortical regions), tumor (> 30%), total body (> 0.1%, urinary bladder excluded), urinary bladder (> 10%). Time courses of activity in blood and total body were analyzed by non-linear regression using the ‘Two-phase decay’ and ‘One-phase decay’ models implemented in Prism 10 (GraphPad Software, San Diego, CA, USA). Total activity amounts in liver and kidneys were estimated using a realistic dosimetry model for 30 g-mice (Keenan et al. 2010).

### Ex vivo biodistribution study

The biodistribution experiments with [^225^Ac]Ac-mcp-A and [^225^Ac]Ac-mcp-AB were performed using A431 tumor-bearing mice. The SCID male mice (ENVIGO, Indianapolis, IN, USA) 8 weeks old were subcutaneously xenografted into the right flank with 1∙10^6^ A431 cells (ATCC, Manassas, VA, USA). The tumor growth was periodically monitored by caliper-based measurements. When the tumor reached a volume of 200–300 mm^3^ (i.e., approx. 3 weeks after xenografting), mice were enrolled in the biodistribution study. All experimental animals were housed in a specific-pathogen-free (SPF) animal facility and all experiments were performed in accordance with appropriate legal norms (Czech Law No. 246/1992) and with the approval of the Ministry of Education, Youth and Sports (MSMT-35035/2019-3) and approval of the Ethical committee of Faculty of Medicine and Dentistry, Palacky University in Olomouc. The number of animals was restricted to *n* = 5 per group and time point in order to strictly follow 3Rs principle.

For biodistribution studies, the ^225^Ac-labeled immunoconjugates were diluted with saline and applied retro-orbitally (r.o.) to the animals at a dose of 50 kBq per mouse corresponding to 0.5 nmol of the sdAbs. Control group was injected with 100 µL of saline. The injections were carried out under 2% isoflurane anesthesia (FORANE, Abbott Laboratories, Abbott Park, IL, USA) to minimize animal suffering and to prevent animal motion. The mice were sacrificed by cervical dislocation 1, 4, 24 and 48 h post-injection and organs of interest (blood, spleen, pancreas, stomach, intestine, kidneys, liver, heart, lungs, muscle, bone, and tumor) were collected. The organs were weighed, and their radioactivity was counted on an automatic gamma counter. Uptake of the radioimmunoconjugates was expressed as a percentage of injected dose per gram of the corresponding organ (% ID/g).

### Histological examination

Tissue samples were fixed in 10% buffered formalin for 24 h and dehydrated in an autotechnicon tissue processor. Afterwards, paraffin blocks were prepared, 0.2 μm tissue sections were obtained using a rotary microtome and stained with hematoxylin and eosin. After evaluating the quality of the samples, immunohistochemically stained slides for γH2AX and Ki67 were made according to the protocol detailed elsewhere (Kurfurstova et al. 2016; Reissig et al. 2022). Slides were scanned at 40x magnification using the Olympus™ Slideview VS200 Research Slide Scanner (UPLXAPO40X (NA 0.95): 0.137 μm/pixel). Following scanning, 3–9 regions (ranging from 57,605 to 533,185 µm^2^) were selected from the scanned tissues with a total cell number of 638–6179 and subsequently analyzed in QuPath-0.4.4 software. Varying the size of the selected regions aimed to exclude artifacts and necrotic areas that could potentially bias the final assessment. A specific workflow approach has been developed in QuPath for the analysis of positive cells in selected areas (for details see Supplemental Information).

## Electronic supplementary material

Below is the link to the electronic supplementary material.

Supplementary Material 1

## Data Availability

The datasets used and/or analyzed during the current study are available from the corresponding author on reasonable request.

## Abbreviations

% ID: Percentage of Injected Dose
AE: Auger Electron
BCN: Bicyclononyne
B_max_: Maximum Binding Capacity
BSA: Bovine Serum Albumin
DMEM: Dulbecco’s Modified Eagle Medium
DPBS: Dulbecco’s Phosphate-Buffered Saline
DOTA: 2,2′,2″,2‴-(1,4,7,10-Tetraazacyclododecane-1,4,7,10-Tetrayl)Tetraacetic Acid
EDTA: Ethylenediaminetetraacetic Acid
EGFR: Epidermal Growth Factor Receptor
ELISA: Enzyme-Linked Immunosorbent Assay
FCS: Fetal Calf Serum
IMAC: Immobilized Metal Affinity Chromatography
iTLC: Instant Thin Layer Chromatography
K_d_: Equilibrium Dissociation Constant
LET: Linear Energy Transfer
mcp: Macropa
MWCO: Molecular Weight Cut-Off
PAGE: Polyacrylamide Gel Electrophoresis
PBS: Phosphate-Buffered Saline
PET: Positron Emission Tomography
PFA: Paraformaldehyde
p.i.: post-injection
rt: room temperature
sdAb: Single-Domain Antibody
SDS: Sodium Dodecyl Sulfate
SG: Silica Gel
SPAAC: Strain-Promoted Azide-Alkyne Cycloaddition
SPECT: Single-Photon Emission Computed Tomography
SPF: Specific-Pathogen-Free
SUV: Standardized Uptake Value
TAT: Targeted Alpha Therapy
TRNT: Targeted Radionuclide Therapy
