# Supplementary material for: It’s a match: use of the radionuclide theranostic pair 133La/225Ac for the radiopharmacological characterization of EGFR-targeted single-domain antibodies
Source: EJNMMI Radiopharm Chem. 2025 Jun 19;10:31. doi: 10.1186/s41181-025-00354-7 (PMC12179020; doi:10.1186/s41181-025-00354-7)
Supplement: Supplementary file 1 — Supplementary Material 1 [file 41181_2025_354_MOESM1_ESM.docx]

**It’s a match: Use of the radionuclide theranostic pair ^133^La/^225^Ac for the radiopharmacological characterization of EGFR-targeted single-domain antibodies**

Johanna Trommer^1^, Martin Ullrich^1^, Falco Reissig^1^, Santiago Andres Brühlmann^1^, Anne-Kathrin Nitt-Weber^1^, Zbynek Novy^2,3^, Katarina Hajduova^2^, Daniela Kurfurstova^4^, Romana Hendrychova^4^, Jan Bouchal^4^, Milos Petrik^2,3,5^, Christin Neuber^1^, Wiebke Sihver^1^, Sven Stadlbauer^1^, Jens Pietzsch^1,6^, Martin Kreller^1^, Klaus Kopka^1,6,7,8^, Constantin Mamat^1,6^, Kristof Zarschler^1*^

^1^ Helmholtz-Zentrum Dresden-Rossendorf, Institute of Radiopharmaceutical Cancer Research, Dresden, Germany.

^2^ Institute of Molecular and Translational Medicine, Faculty of Medicine and Dentistry, Palacký University, Olomouc, Czech Republic.

^3^ Czech Advanced Technology and Research Institute, Palacký University, Olomouc, Czech Republic.

^4^ Institute of Clinical and Molecular Pathology, Faculty of Medicine and Dentistry, Palacký University Olomouc, Czech Republic.

^5^ Laboratory of Experimental Medicine, University Hospital, Olomouc, Czech Republic.

^6^ Technische Universität Dresden, School of Science, Faculty of Chemistry and Food Chemistry, Dresden, Germany.

^7^ National Center for Tumor Diseases (NCT) Dresden, University Hospital Carl Gustav Carus, Fetscherstraße 74, 01307, Dresden, Germany.

^8^ German Cancer Consortium (DKTK), Partner Site Dresden, Fetscherstraße 74, 01307, Dresden, Germany.

**Content**

| 1. | Synthesis, ^1^H and ^13^C NMR spectra of compounds | ……...………… | 2 |
| --- | --- | --- | --- |
| 2. | Radio-TLC chromatograms of ^225^Ac-labeled sdAbs | ……...………… | 11 |
| 3. | Biological data | ……...………… | 11 |
| 4. | Scanning parameters of histological specimens | ……...………… | 12 |
| 5. | QuPath parameters | ……...………… | 12 |
| 6. | Biodistribution data of [^225^Ac]Ac-mcp-A and [^225^Ac]Ac-mcp-AB | ……...………… | 16 |

**1. Synthesis, ^1^H and ^13^C NMR spectra of compounds**

**Dimethyl 4-((14-azido-3,6,9,12-tetraoxatetradecyl)oxy)pyridine-2,6-dicarboxylate (3)**

**Supplemental figure 1:** Synthesis pathway for compound **3**.

14-Azido-3,6,9,12-tetraoxatetradecyl tosylate (**1**, 420 mg, 1.01 mmol), dimethyl 4-hydroxypyridine-2,6-dicarboxylate (**2**, 319 mg, 1.51 mmol), and K_2_CO_3_ (529 mg, 3.83 mmol) were dissolved in anhydrous acetonitrile (10 mL) and stirred at 85 °C overnight. After cooling to room temperature, the solids were filtered, the solvent was removed and the crude product was purified via automated column chromatography (chloroform: ethyl acetate = 10:1) to yield compound **3** (190 mg, 41%) as colorless syrup. ^1^H NMR (400 MHz, CDCl_3_): δ = 3.38 (t, ^3^*J* = 5.1 Hz, 2H, CH_2_), 3.64–3.69 (m, 12 H, OCH_2_), 3.70–3.74 (m, 2H, OCH_2_), 3.90 (t, ^3^*J* = 4.6 Hz, 2H, CH_2_), 4.00 (s, 6H, CH_3_), 4.31 (t, ^3^*J* = 4.6 Hz, 2H, CH_2_), 7.83 (s, 2H, Ar-H) ppm; ^13^C NMR (101 MHz, CDCl_3_): δ = 50.8 (CH_2_), 53.4 (CH_3_), 68.6, 69.2, 70.2, 70.7, 70.8, 70.8, 70.9, 71.1 (8 x CH_2_), 114.8 (CH_Ar_), 149.9 (C_Ar_), 165.3 (C_Ar_), 167.1 (C=O) ppm. MS (ESI+): *m/z* = 457 [M+H]^+^, 479 [M+Na]^+^.

**Supplemental figure 2:** ^1^H NMR spectrum of compound **3**.

**Supplemental figure 3:** ^13^C NMR spectrum of compound **3**.

**Methyl 4-((14-azido-3,6,9,12-tetraoxatetradecyl)oxy)-6-(hydroxymethyl)picolinate (4)**

**Supplemental figure 4:** Synthesis pathway for compound **4**.

Compound **3** (385 mg, 0.84 mmol) was dissolved in anhydrous methanol (10 mL) and NaBH_4_ (32 mg, 0.84 mmol) was added. The mixture was stirred for 5 h at 65 °C. Afterwards, the solvent was removed, saturated NH_4_Cl solution was added, the aqueous phase extracted with chloroform (3 x 10 mL). The combined organic phases were dried over Na_2_SO_4_, the solvent was removed and purification was done via automated column chromatography (chloroform:ethyl acetate = 10:1) to yield compound **4** (190 mg, 53%) as colorless syrup. ^1^H NMR (400 MHz, CDCl_3_): δ = 3.37 (t, ^3^*J* = 5.0 Hz, 2H, CH_2_), 3.62–3.68 (m, 12 H, OCH_2_), 3.68–3.71 (m, 2H, OCH_2_), 3.87 (t, ^3^*J* = 4.7 Hz, 2H, CH_2_), 3.96 (s, 3H, CH_3_), 4.24 (t, ^3^*J* = 4.6 Hz, 2H, CH_2_), 4.78 (s, 3H, CH_3_), 7.09 (d, ^4^*J* = 2.3 Hz, 1H, Ar-H), 7.56 (d, ^4^*J* = 2.3 Hz, 1H, Ar-H) ppm; ^13^C NMR (101 MHz, CDCl_3_): δ = 50.8 (CH_2_), 53.1 (CH_3_), 64.8, 68.1, 69.4, 70.1, 70.7, 70.7, 70.8, 70.8, 70.9, 71.1 (10 x CH_2_), 109.8 (CH_Ar_), 111.3 (CH_Ar_), 148.6 (C_Ar_), 162.4 (C=O), 165.6 (C_Ar_), 166.8 (C_Ar_) ppm. MS (ESI+): *m/z* = 429 [M+H]^+^, 451 [M+Na]^+^.

**Supplemental figure 5:** ^1^H NMR spectrum of compound **4**.

**Supplemental figure 6:** ^13^C NMR spectrum of compound **4**.

**Methyl 4-((14-azido-3,6,9,12-tetraoxatetradecyl)oxy)-6-(chloromethyl)picolinate (5)**

**Supplemental figure 7:** Synthesis pathway for compound **5**.

Compound **4** (140 mg, 0.33 mmol) was cooled to 0 °C, SOCl_2_ (1 mL) was slowly added and the mixture was stirred at 0 °C for 1 h and at room temperature for 2 h. Afterwards, the mixture was added dropwise to an ice-cold saturated hydrogen carbonate solution (15 mL). The aqueous solution was extracted with dichloromethane (3 x 15 mL) to yield compound **5** (120 mg, 82%) as a colorless syrup without further purification. ^1^H NMR (400 MHz, CDCl_3_): δ = 3.37 (t, ^3^*J* = 4.5 Hz, 2H, CH_2_), 3.62–3.68 (m, 12 H, OCH_2_), 3.69–3.74 (m, 2H, OCH_2_), 3.89 (t, ^3^*J* = 4.2 Hz, 2H, CH_2_), 3.99 (s, 3H, CH_3_), 4.26 (t, ^3^*J* = 4.1 Hz, 2H, CH_2_), 4.71 (s, 3H, CH_3_), 7.25 (br s, 1H, Ar-H), 7.62 (br s, 1H, Ar-H) ppm; ^13^C NMR (101 MHz, CDCl_3_): δ = 46.4 (CH_2_), 50.8 (CH_2_), 53.1 (CH_3_), 68.2, 69.3, 70.2, 70.7, 70.8, 70.8, 70.8, 71.1 (8 x CH_2_), 111.5 (CH_Ar_), 112.6 (CH_Ar_), 149.2 (C_Ar_), 158.9 (C=O), 165.5 (C_Ar_), 166.9 (C_Ar_) ppm. MS (ESI+): *m/z* = 447 [M+H; ^35^Cl]^+^, 449 [M+H; ^37^Cl]^+^, 469 [M+Na; ^35^Cl]^+^, 471 [M+H; ^37^Cl]^+^.

**Supplemental figure 8:** ^1^H NMR spectrum of compound **5**.

**Supplemental figure 9:** ^13^C NMR spectrum of compound **5**.

**Methyl 6-((16-(3-((14-azido-3,6,9,12-tetraoxatetradecyl)oxy)-5-(methoxycarbonyl)benzyl)-1,4,10,13-tetraoxa-7,16-diazacyclooctadecan-7-yl)methyl)picolinate (7)**

**Supplemental figure 10:** Synthesis pathway for compound **7**.

Compound **5** (105 mg, 0.23 mmol), methyl 6-((1,4,10,13-tetraoxa-7,16-diazacyclooctadecan-7-yl)methyl)-picolinate (**6**, 194 mg, 0.47 mmol), diisopropylethylamine (0.5 mL), and NaI (approx. 10 mg) were dissolved in anhydrous acetonitrile (10 mL) and the mixture was stirred at 85 °C overnight. After TLC control, the solvent was removed and the crude product was purified via automated column chromatography (ethyl acetate: ethanol = 1:0 → 0:1) to yield compound **7** (192 mg, 99%) as colorless syrup. ^1^H NMR (400 MHz, CDCl_3_): δ = 3.38 (t, ^3^*J* = 5.0 Hz, 2H, CH_2_), 3.61–3.73 (m, 34 H, OCH_2_), 3.85–4.00 (m, 18H, 2 x CH_3_+OCH_2_), 4.27 (t, ^3^*J* = 3.9 Hz, 2H, CH_2_), 4.68 (s, 3H, CH_3_), 4.78 (s, 3H, CH_3_), 7.34 (br s, 1H, Ar-H), 7.65 (br s, 1H, Ar-H), 7.76 (d, ^3^*J* = 7.7 Hz, 1H, Ar-H), 7.96 (t, ^3^*J* = 7.7 Hz, 1H, Ar-H), 8.12 (d, ^3^*J* = 7.7 Hz, 1H, Ar-H) ppm; ^13^C NMR (101 MHz, CDCl_3_): δ = 50.8 (CH_2_), 53.2 (CH_3_), 54.6, 57.1, 57.3, 65.1, 65.2, 68.2, 69.2, 70.1, 70.4, 70.5, 70.6, 70.7, 70.7, 70.9 (8 x CH_2_), 113.4 (CH_Ar_), 113.9 (CH_Ar_), 125.6 (CH_Ar_), 128.4 (CH_Ar_), 139.2 (CH_Ar_), 147.8 (C_Ar_), 149.0 (C_Ar_), 150.6 (C=O), 152.0 (C=O), 164.8 (C_Ar_), 167.4 (C_Ar_) ppm. MS (ESI+): *m/z* = 411 [M+2H]^2+^, 822 [M+H]^+^, 860 [M+K]^+^.

**Supplemental figure 11:** ^1^H NMR spectrum of compound **7**.

**Supplemental figure 12:** ^13^C NMR spectrum of compound **7**.

**6-((16-(3-((1-(Bicyclo[6.1.0]non-4-yn-9-yl)-3-oxo-2,7,10,13,16-pentaoxa-4-azaoctadecan-18-yl)oxy)-5-carboxybenzyl)-1,4,10,13-tetraoxa-7,16-diazacyclooctadecan-7-yl)methyl)picolinic acid (BCN-PEG_5_-mcp)**

**Supplemental figure 13:** Synthesis pathway for **BCN-PEG_5_-mcp**.

Compound **7** (90 mg, 0.11 mmol) was dissolved in methanol (5 mL) and Pd/C (20 mg) was added. The flask was flushed with H_2_ and stirred vigorously at room temperature overnight under H_2_ atmosphere. After HPLC control, the mixture was filtered, the solvent was removed and the yielded product was used without purification (80 mg, 92%) as colorless syrup. Afterwards, the amine (80 mg, 0.10 mmol) was dissolved in a mixture of methanol and water (3 mL, v:v = 1:1), LiOH (14 mg) was added and the mixture was stirred at room temperature for 3 h. After HPLC control, the solvent was removed and the crude product was purified via semipreparative HPLC (gradient: 10% → 30% ACN in water + 0.1 % TFA) to give the deprotected compound (35 mg, 45%) as colorless syrup after lyophilization. Finally, the deprotected compound (35 mg, 0.05 mmol), exo-BCN-pNPE (16 mg, 0.05 mmol), and Et_3_N (20 µL) were dissolved in anhydrous acetonitrile (2 mL) and stirred at room temperature overnight. Afterwards, the solvent was removed and crude product was purified via semipreparative HPLC (gradient: 10% → 30% ACN in water + 0.1 % TFA) to give **BCN-PEG_5_-mcp** (15 mg, 35%; 15% over 3 steps) as colorless syrup after lyophilization. ^1^H NMR (400 MHz, CD_3_CN): δ = 0.57–0.74 (m, 3H, BCN), 1.26–1.40 (m, 2H, BCN), 2.08 (br. d, ^2^*J* = 15.8 Hz, 2H, BCN), 2.17–2.37 (m, 4H, BCN), 3.21 (q, ^3^*J* = 5.6 Hz, CH_2_), 3.41–3.66 (m, 30H, CH_3_+CH_2_), 3.79–3.93 (m, 12H, CH_2_), 4.31 (t, ^3^*J* = 4.2 Hz, 3H, CH_2_), 4.64 (br. s, 2H, CH_2_Ar), 4.78 (br. s, 2H, CH_2_Ar), 5.62 (br. s, 1H, NH), 7.20 (s, 1H, Ar-H), 7.62–7.69 (m, 2H, Ar-H), 8.05 (t, ^3^*J* = 7.7 Hz, 1H, Ar-H), 8.13 (d, ^3^*J* = 7.7 Hz, Ar-H) ppm; ^13^C NMR (101 MHz, CDCl_3_): δ = 21.8, 23.6, 24.7 , 34.1 (4 x BCN), 41.5 (CH_2_), 55.2 (BCN), 58.1, 58.5, 66.2, 66.3, 69.3, 69.7, 69.8, 70.5, 70.8, 70.9, 71.1, 71.2, 71.4 (x CH_2_), 99.7 (C≡C), 111.2, 114.1, 124.9, 128.0, 140.6 (5 x CH_Ar_), 148.2, 149.9, 157.7 (C_Ar_) 165.7 (C=O), 166.2 (C=O), 168.9 (C=O_BCN_) ppm. MS (ESI+): *m/z* = 944 [M+H]^+^, 966 [M+Na]^+^.

**Supplemental figure 14:** ^1^H NMR spectrum of **BCN-PEG_5_-mcp.**

**Supplemental figure 15:** ^13^C NMR spectrum of **BCN-PEG_5_-mcp**.

**2. Radio-TLC chromatograms of ^225^Ac-labeled sdAbs**


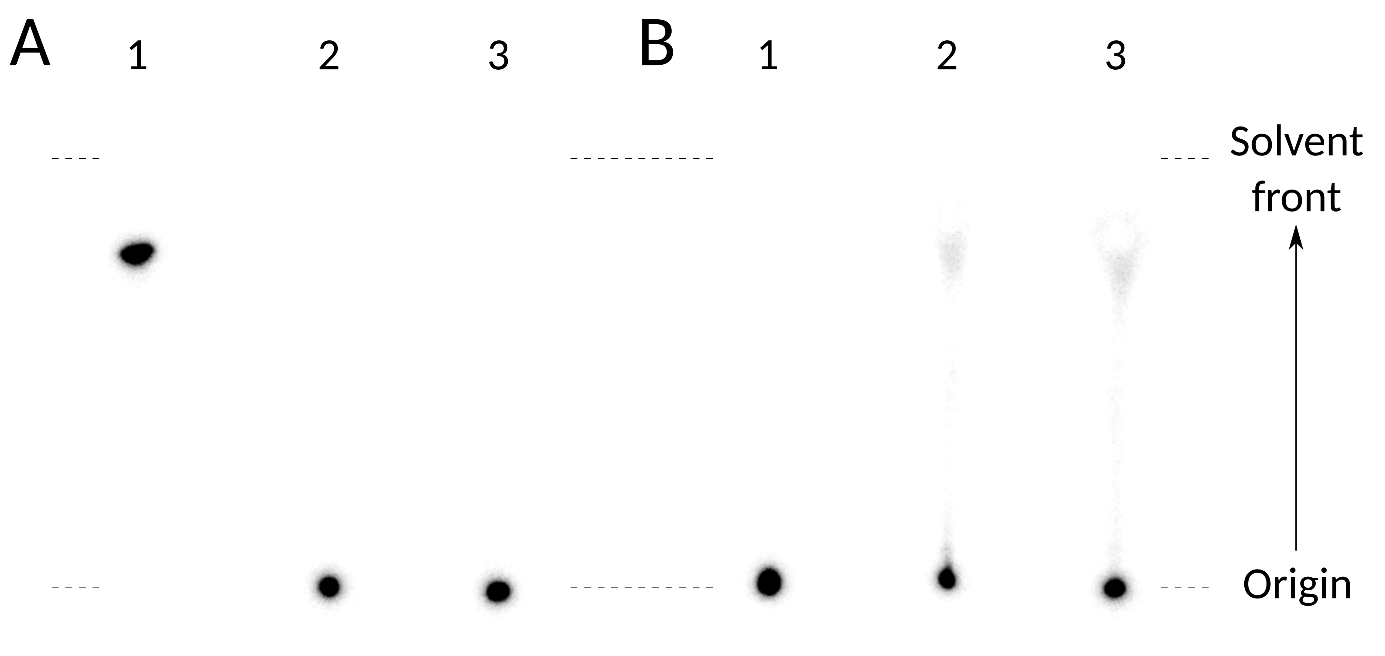


**Supplemental figure 16:** Radio-TLC of ^225^Ac-labeled, macropa-conjugated sdAbs. In the normal phase TLC system (A), [^225^Ac]Ac-mcp-A (2) and [^225^Ac]Ac-mcp-AB (3) remain at the origin (R_f_ = 0), whereas free [^225^Ac]Ac (1) is complexed by EDTA of the mobile phase and migrates as [^225^Ac]Ac-EDTA with the solvent front (R_f_ = 1). The reverse phase TLC system (B) was designed to detect traces of radiolabeled **BCN-PEG_5_-mcp** with an R_f_ ~0.7, whereas free [^225^Ac]Ac^3+^ (1), [^225^Ac]Ac-mcp-A (2) and [^225^Ac]Ac-mcp-AB (3) remain at the origin. The residual traces of radiolabeled **BCN-PEG_5_-mcp** were removed by spin filtration prior to further use.

**3. Biological data**


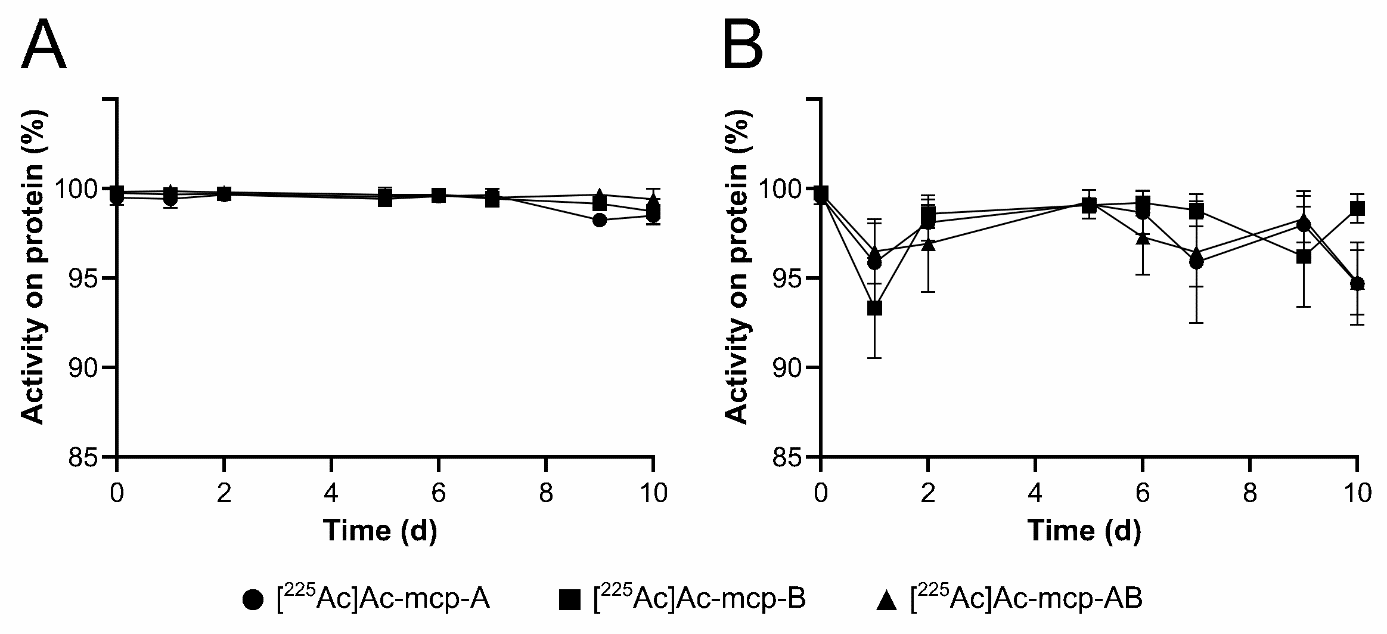


**Supplemental figure 17:** Time-dependent stability examination of ^225^Ac-labeled macropa-functionalized immunoconjugates over a period of up to 10 d in PBS at room temperature (A) and human serum at 37 °C (B).


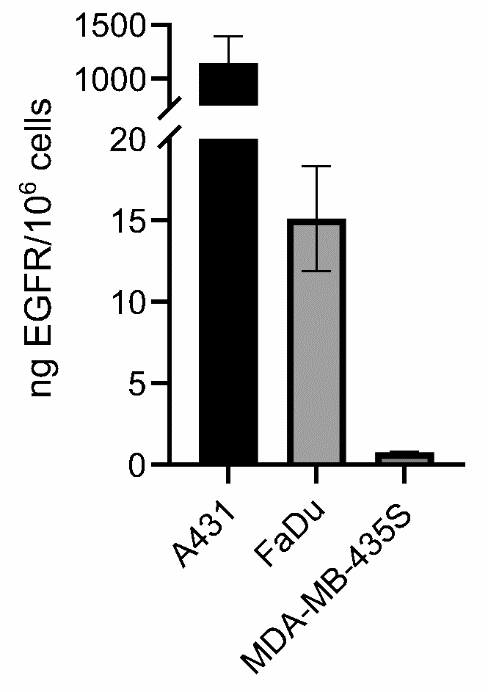


**Supplemental figure 18:** Analysis of EGFR expression level using lysates of indicated human cell lines. The amount of full-length human EGFR is expressed as quantity (ng) per 1 million cells.

**4. Scanning parameters of histological specimens (γH2AX, Ki67)**

- Magnification: 40x
- Focus: Ultra-high
- Number of selections per one scan tissue: 3-9

**5. QuPath parameters**

**a) QuPath parameters for kidney sections - γH2AX staining**

1. *Designation of the whole area*
2. *Analyze → Positive cell detection*:

Setup parameters:

- Detection image: Optical density sum
- Requested pixel size: 0.5 μm

Nucleus parameters:

- Background radius: 50 μm
- Use opening by reconstruction
- Median filter radius: 0 μm
- Sigma: 1.5 μm
- Minimum area: 5 μm^2
- Maximum area: 400 μm^2

Intensity parameters:

- Threshold: 0.07
- Max background intensity: 2
- Split by shape

Cell parameters:

- Cell expansion: 5 μm
- Include cell nucleus

General parameters:

- Smooth boundaries
- Make measurements

Intensity threshold parameters:

- Score compartment: Nucleus: DAB OD max
- Threshold 1+ :0.13
- Threshold 2+ :0.4
- Threshold 3+ :0.6
- Single threshold

**b) QuPath parameters for tumor sections - γH2AX staining**

1. *Designation of the whole area*
2. *Analyze → Positive cell detection*:

Setup parameters:

- Detection image: Optical density sum
- Requested pixel size: 0.5 μm

Nucleus parameters:

- Background radius: 50 μm
- Use opening by reconstruction
- Median filter radius: 0 μm
- Sigma: 1.5 μm
- Minimum area: 5 μm^2
- Maximum area: 400 μm^2

Intensity parameters:

- Threshold: 0.07
- Max background intensity: 2
- Split by shape

Cell parameters:

- Cell expansion: 5 μm
- Include cell nucleus

General parameters:

- Smooth boundaries
- Make measurements

Intensity threshold parameters:

- Score compartment: Nucleus: DAB OD max
- Threshold 1+ :0.08
- Threshold 2+ :0.4
- Threshold 3+ :0.6
- Single threshold

**c) QuPath parameters for tumor sections – Ki67 staining**

1. *Designation of the whole area*
2. *Analyze → Positive cell detection*:

Setup parameters:

- Detection image: Optical density sum
- Requested pixel size: 0.5 μm

Nucleus parameters:

- Background radius: 20 μm
- Use opening by reconstruction
- Median filter radius: 0 μm
- Sigma: 1.5 μm
- Minimum area: 10 μm^2
- Maximum area: 400 μm^2

Intensity parameters:

- Threshold: 0.1
- Max background intensity: 2
- Split by shape

Cell parameters:

- Cell expansion: 5 μm
- Include cell nucleus

General parameters:

- Smooth boundaries
- Make measurements

Intensity threshold parameters:

- Score compartment: Nucleus: DAB OD mean
- Threshold 1+ :0.4
- Threshold 2+ :0.4
- Threshold 3+ :0.6
- Single threshold

**6. Biodistribution data of [^225^Ac]Ac-mcp-A and [^225^Ac]Ac-mcp-AB**

**Supplemental table 1:** Quantitative evaluation of [^225^Ac]Ac-mcp-A in A431 tumor-bearing mice. The values are given as the mean of the percentage of injected dose per gram of organ ± standard deviation (n = 5).

| **Organ** | **1 h p.i.** | | **4 h p.i.** | | **24 h p.i.** | | **48 h p.i.** | |
| --- | --- | --- | --- | --- | --- | --- | --- | --- |
|  | **Mean** | **SD** | **Mean** | **SD** | **Mean** | **SD** | **Mean** | **SD** |
| Blood | 0.83 | 0.10 | 0.48 | 0.04 | 0.11 | 0.01 | 0.11 | 0.01 |
| Spleen | 1.51 | 0.39 | 1.26 | 0.18 | 1.02 | 0.39 | 0.55 | 0.05 |
| Pancreas | 0.24 | 0.03 | 0.20 | 0.02 | 0.09 | 0.02 | 0.08 | 0.03 |
| Stomach | 0.35 | 0.03 | 0.34 | 0.04 | 0.14 | 0.02 | 0.19 | 0.16 |
| Intestine | 0.40 | 0.06 | 0.52 | 0.06 | 0.23 | 0.11 | 0.28 | 0.15 |
| Kidneys | 149.09 | 23.54 | 101.38 | 6.67 | 72.68 | 7.04 | 32.25 | 3.67 |
| Liver | 3.29 | 0.74 | 4.54 | 0.45 | 2.57 | 0.46 | 1.95 | 0.23 |
| Heart | 0.40 | 0.07 | 0.47 | 0.02 | 0.13 | 0.04 | 0.15 | 0.03 |
| Lung | 1.71 | 0.38 | 1.56 | 0.79 | 0.34 | 0.16 | 0.70 | 0.57 |
| Muscle | 0.14 | 0.03 | 0.09 | 0.03 | 0.05 | 0.04 | 0.05 | 0.01 |
| Femur | 0.48 | 0.14 | 0.74 | 0.27 | 0.57 | 0.10 | 0.33 | 0.16 |
| Tumor | 0.55 | 0.10 | 0.39 | 0.08 | 0.28 | 0.02 | 0.28 | 0.02 |

**Supplemental table 2:** Quantitative evaluation of [^225^Ac]Ac-mcp-AB in A431 tumor-bearing mice. The values are given as the mean of the percentage of injected dose per gram of organ ± standard deviation (n = 5).

| **Organ** | **1 h p.i.** | | **4 h p.i.** | | **24 h p.i.** | | **48 h p.i.** | |
| --- | --- | --- | --- | --- | --- | --- | --- | --- |
|  | **Mean** | **SD** | **Mean** | **SD** | **Mean** | **SD** | **Mean** | **SD** |
| Blood | 1.50 | 0.29 | 0.57 | 0.09 | 0.15 | 0.04 | 0.06 | 0.01 |
| Spleen | 2.20 | 0.48 | 3.39 | 1.11 | 1.10 | 0.41 | 0.78 | 0.27 |
| Pancreas | 0.37 | 0.04 | 0.48 | 0.08 | 0.14 | 0.06 | 0.09 | 0.01 |
| Stomach | 0.33 | 0.07 | 0.52 | 0.10 | 0.14 | 0.07 | 0.14 | 0.03 |
| Intestine | 0.62 | 0.10 | 1.45 | 0.35 | 0.18 | 0.04 | 0.19 | 0.05 |
| Kidneys | 154.58 | 30.71 | 121.66 | 25.59 | 102.23 | 31.97 | 47.72 | 11.80 |
| Liver | 2.47 | 0.48 | 3.53 | 0.82 | 2.21 | 0.45 | 1.66 | 0.23 |
| Heart | 0.66 | 0.10 | 0.56 | 0.13 | 0.20 | 0.04 | 0.13 | 0.05 |
| Lung | 1.43 | 0.26 | 0.90 | 0.39 | 0.40 | 0.15 | 0.32 | 0.30 |
| Muscle | 0.16 | 0.06 | 0.14 | 0.03 | 0.09 | 0.03 | 0.04 | 0.01 |
| Femur | 0.46 | 0.15 | 0.51 | 0.09 | 0.29 | 0.14 | 0.32 | 0.21 |
| Tumor | 0.87 | 0.12 | 0.59 | 0.12 | 0.58 | 0.14 | 0.36 | 0.03 |
